# Peptides, not vitamins: Yeast extract components sustaining glucose respiration of the halotolerant acidophile *Sulfobacillus thermosulfidooxidans*

**DOI:** 10.1007/s11274-026-05336-8

**Published:** 2026-11-05

**Authors:** Katarzyna Kwiecień, Stefan Kaschabek, Jan Zuber, Michael Schlömann

**Affiliations:** 1 Environmental Microbiology, Institute of Biosciences, TU Bergakademie Freiberg, Lessingstraße 45, 09599 Freiberg, Germany; 2 Institute for Analytical Chemistry, TU Bergakademie Freiberg, Lessingstraße 45, 09599 Freiberg, Germany

**Keywords:** Chloride tolerance, Halotolerance, Mass spectrometry, Peptides, Respiratory activity, *Sulfobacillus thermosulfidooxidans*, Yeast extract

## Abstract

The use of *Sulfobacillus thermosulfidooxidans* DSM 9293^T^ in bioleaching of sulfidic ores under saline conditions is limited by chloride-induced stress, despite some inherent halotolerance of the species. While yeast extract supplementation can improve chloride tolerance, its undefined composition hinders process standardization. To address this, we systematically investigated which components of yeast extract are essential for supporting respiration of *Sulfobacillus thermosulfidooxidans*, under assay conditions without additional NaCl, aiming to replace it with compositionally simpler medium supplementation. Our comparative assessments confirmed *Sulfobacillus thermosulfidooxidans* as the most halotolerant among the tested *Sulfobacillus* species, tolerating up to 500 mM NaCl (approximately 17.7 g Cl⁻ l⁻¹) while maintaining Fe(II)-oxidizing activity. Respiration assays revealed that casamino acids (CA), glucose, and potassium supported substantial respiratory activity and reached an endpoint comparable to that obtained with YE by day 7, although with a delayed onset, whereas vitamins alone had minimal effect. Further omission experiments identified the glutamate, aspartate, histidine, and alanine/branched-chain amino acid groups as important contributors to respiration. Molecular weight fractionation indicated that yeast extract fractions between 0.5 and 1 kDa primarily stimulated respiration. Separately, FT-ICR-MS analysis of CA-derived material identified 118 unique elemental formulas compatible with di- or tripeptide compositions, of which 26 showed culture-associated disappearance relative to uninoculated controls. Although high-resolution FT-ICR-MS provides accurate elemental compositions, without fragmentation data it cannot distinguish between isomeric peptide sequences or isobaric residues sharing the same molecular formula. Therefore, these assignments were interpreted as candidate peptide compositions rather than sequence-resolved peptides. Together, these findings demonstrate that CA, glucose, and potassium can sustain glucose-dependent respiration to an endpoint comparable to YE, although with slower respiratory activation. CA nevertheless remains chemically complex and therefore represents a compositionally simpler rather than fully defined supplementation strategy. Whether this supplementation provides comparable support under chloride stress remains to be tested directly.

**Supplementary Information:**

The online version contains supplementary material available at https://doi.org/10.1007/s11274-026-05336-8.

## Key Points

In contrast to other *Sulfobacillus* species *Sulfobacillus thermosulfidooxidans* tolerates up to 500 mM NaCl while retaining Fe(II)-oxidizing activity.

Several different compounds seem to contribute to glucose respiration by *Sb. thermosulfidooxidans*.

The 0.5 -1 kDa mass fraction of yeast extract showed the highest stimulation of respiratory activity with glucose.

Casamino acids, glucose, and potassium sustain metabolic activity with delayed onset but an endpoint comparable to YE by day 7.

**Supplementary Information:**

The online version contains supplementary material available at https://doi.org/10.1007/s11274-026-05336-8.

## Introduction

Biohydrometallurgy, which utilizes iron- and sulfur-oxidizing bacteria for the recovery of metals from low-grade sulfide ores, offers, under certain conditions, an environmentally sustainable and economically attractive alternative to conventional pyrometallurgical processes (Nkuna et al. 2022; Erüst et al. 2013). In industrial-scale operations, bioleaching may face challenges related to chloride accumulation in recirculating leachates, particularly where evaporation concentrates salts within the system or in regions relying on saline or seawater sources for processing, or rich in atacamite (Martins and Leão 2023). Therefore, in recent years, interest has grown in bioleaching processes conducted under elevated chloride concentrations, which are also known to increase the efficiency of chalcopyrite leaching by destabilization of passivation layers and facilitation of mineral dissolution (Chen and Schlömann 2023; Watling et al. 2016; Bevilaqua et al. 2013; Chang-Li et al. 2012; Yoo et al. 2010). However, high chloride levels impose significant physiological stress on microbial communities, including osmotic, oxidative, and acid stress (Rivera-Araya et al. 2019). Chloride ions under acidic conditions are to some extent membrane-permeable and can collapse the proton motive force in acidophilic bacteria, leading to loss of energy conservation and disruption of pH homeostasis within the cytoplasm, conditions that are detrimental to cell survival and metabolic activity (Suzuki et al. 1999; Rivera-Araya et al. 2019). This sensitivity limits the effectiveness of acidophilic iron- and sulfur-oxidizers under saline conditions and necessitates the development of strategies to enhance microbial halotolerance without compromising biooxidative activity. Moreover, this challenge underscores the importance of identifying or engineering strains that can tolerate such conditions while maintaining biooxidative activity.

*Sulfobacillus thermosulfidooxidans*, a Gram-positive, moderately thermophilic, mixotrophic acidophile, has demonstrated the capacity to oxidize both ferrous iron and reduced inorganic sulfur compounds under acidic conditions, rendering it suitable for bioleaching applications in tanks and heaps (Karavaiko et al. 2005; Norris et al. 1996). Previous studies have revealed the presence of genes in *Sb. thermosulfidooxidans* to potentially cope with increased NaCl concentrations and reported its ability to tolerate NaCl concentrations ranging up to 500 mM depending on prior adaptation, growth substrate, and medium composition (Rivera-Araya et al. 2017, 2020; Huynh et al. 2020a; b). Prior studies on acidophilic bacteria have demonstrated that chloride ions inhibit iron oxidation more strongly than sulfur oxidation (Harahuc et al. 2000). However, this aspect has not been systematically addressed for moderately thermophilic mixotrophs such as *Sb. thermosulfidooxidans*. While *Sb. thermosulfidooxidans* exhibits higher tolerance to chloride compared to many other acidophilic iron oxidizers, detailed investigations into the metabolic pathways and nutrient requirements that support this halotolerance remain limited (Rivera-Araya et al. 2020).

Yeast extract (YE), a complex nutrient supplement, has been shown to enhance the activity and chloride tolerance of *Sb. thermosulfidooxidans* under saline conditions (Huynh et al. 2020a; b). However, its chemically complex and batch-dependent composition complicates mechanistic interpretation and may limit reproducibility between experiments and laboratories. YE contains a broad range of peptides, amino acids, vitamins, nucleotides, and inorganic constituents that may contribute differently to cellular metabolism (Mosser et al. 2012, 2015). However, which of these constituents are responsible for supporting respiration of *Sb. thermosulfidooxidans* remains poorly resolved. Identifying such components would therefore provide a basis for developing compositionally simpler supplementation strategies and for distinguishing general nutritional requirements from responses that are specifically induced by chloride stress.

To address these questions, the present study had two complementary aims. First, chloride tolerance was compared among several *Sulfobacillus* species to determine the relative chloride tolerance of *Sb. thermosulfidooxidans* DSM 9293T within the genus *Sulfobacillus*. Second, the nutritional components supporting glucose-dependent respiration of *Sb. thermosulfidooxidans* were investigated under assay conditions without additional NaCl, with particular emphasis on amino acids, potassium, vitamins, low-molecular-weight fractions of YE, and compounds present in casamino acids. Together, these experiments characterize two distinct aspects of *Sb. thermosulfidooxidans* physiology: its relative NaCl tolerance within the genus and the nutritional factors supporting respiration in the absence of added NaCl.

## Materials and methods

### Determination of chloride tolerance

To assess the chloride tolerance across different *Sulfobacillus* species, five type strains (*Sb. harzensis* DSM 109850^T^, *Sb. sibiricus* DSM 17363^T^, *Sb. acidophilus* DSM 10332^T^, *Sb. thermosulfidooxidans* DSM 9293^T^, and *Sb. benefaciens* DSM 19468^T^, obtained from Deutsche Sammlung von Mikroorganismen und Zellkulturen, DSMZ) were cultivated in modified Mackintosh (1978) medium (initial pH 1.8), supplemented with 5.56 g l⁻¹ (20 mM) FeSO₄·7 H₂O, 220 mg l^-1^ CoCl_2_*6 H_2_O, and 0.2 g l⁻¹ YE, with the exception of *Sb. sibiricus*, which required a higher iron concentration (44.48 g l⁻¹, 160 mM Fe²⁺). These type strains were selected based on their availability in our laboratory and their ability to establish actively growing cultures under the experimental conditions used. The resulting strain panel was therefore intended as a comparative selection rather than a comprehensive survey of all *Sulfobacillus* species. Each culture was inoculated with 10% (v/v) of actively growing precultures grown in the corresponding modified Mackintosh medium without added NaCl and incubated in 6-well plates at 45 °C (or 37 °C for *Sb. benefaciens*) with shaking at 120 rpm. A common assay temperature of 45 °C was used for the four moderately thermophilic strains to provide a largely standardized basis for comparison under the conditions employed in this study, whereas *Sb. benefaciens* was incubated at 37 °C because of its lower temperature optimum (Zhang et al. 2021). The assay was not designed to determine the maximum chloride tolerance of each species at its individual optimum growth temperature.

Sodium chloride concentrations ranging from 0 to 1000 mM were tested, initially in 100 mM increments. Based on initial findings, the concentration steps were subsequently refined to 25 mM intervals around the inhibition threshold to more precisely determine the chloride-tolerance threshold. Bacterial activity was monitored via visual assessment of iron oxidation, as indicated by a shift in medium color from pale green to orange-brown. Chloride tolerance was defined operationally as the highest NaCl concentration at which biological Fe(II) oxidation remained detectable under the tested conditions.

### Bacteria and culture media

*Sb. thermosulfidooxidans* was cultivated in modified Mackintosh (1978) basic salt solution (pH 1.8), supplemented with 2.78 g l⁻¹ (10 mM) FeSO₄·7 H₂O, 220 mg l⁻¹ CoCl₂·6 H₂O, and 0.2 g l⁻¹ YE, depending on the experimental conditions. The YE used in this study was BioChemica yeast extract (AppliChem, product number: A1552, 1000, lot number: 3W001245). The same lot was used throughout all experiments reported in this study. A low initial concentration of ferrous iron was selected for preculture growth based on previous findings (Huynh et al. 2024) with other bacteria, which demonstrated that higher Fe²⁺ concentrations (e.g., 25 mM or 50 mM) often resulted in incomplete oxidation, even during extended incubation periods. Cultures of *Sb. thermosulfidooxidans* were grown in shaking flasks at 45 °C and 120 rpm until cells reached the exponential phase. Cell density was monitored microscopically using a Neubauer counting chamber (Paul Marienfeld, 0.02 mm depth), and harvesting was performed when cultures reached approximately 2 × 10⁷ cells ml⁻¹.

10% (v/v) inoculum from an approximately 72-hour pre-culture was washed three times by centrifugation at 10,000 rpm with sterile, iron-free modified Mackintosh (1978) medium (pH 1.8) to minimize carryover of ferrous/ferric iron and residual nutrients from the preculture. The final pellet was resuspended in freshly prepared medium supplemented with the respective experimental components, according to the downstream application.

### Respiratory activity assessment

The experimental setup for oxygen saturation monitoring was based on the methodology previously described by Kwiecień et al. (2025), with sensor spot-based detection in sealed serum bottles and normalization of values relative to initial O₂ saturation levels.

Measurements were performed at 45 °C using the OxygenLogger software until oxygen readings stabilized. The change in percentage of oxygen saturation was used as a proxy for respiratory activity. A value of 100% therefore represents the normalized starting point, whereas progressively lower values indicate increasing biological oxygen consumption. Because the initial readings occasionally exceeded atmospheric saturation, likely as a consequence of equilibration or measurement effects immediately after preparation of the assay, individual datasets were normalized to the first stabilized measurement (t1), which was set to 100% for each replicate. Throughout the manuscript, the resulting parameter is referred to as normalized O₂ saturation remaining (%). *Assessment of the combined effect of amino acids*,* vitamins*,* and potassium on respiratory activity*.

To investigate the effect of selected growth-promoting components, *Sb. thermosulfidooxidans* was cultivated in defined iron-free modified Mackintosh (1978) medium (pH 1.8) supplemented with 0.5 g l⁻¹ glucose. Various combinations and concentrations of Casamino Acids (casein hydrolysate, Carl Roth 2021, product number Art.-Nr.: A157.2, lot number/ charge throughout the experiments: 500300038) (0.3 g l^− 1^ and 0.6 g l^− 1^), a mixture of 6 vitamins with the concentrations corresponding to the YE AppliChem brochure (0.022 µg ml^− 1^ thiamine, 0.020 µg ml^− 1^ riboflavin, 0.032 µg ml^− 1^ pantothenic acid, 0.014 µg ml^− 1^ pyridoxine, 0.2 µg ml^− 1^ niacin, and 3*10^− 4^ µg ml^− 1^ cyanocobalamin) and doubled concentrations, and potassium sulfate (0.03 g l^− 1^ and 0.06 g l^− 1^) were tested. The same lot of each commercial preparation was used throughout all experiments reported in this study.

Cultures were inoculated with 10% (v/v) of an exponentially growing preculture and incubated in tightly sealed serum bottles at 45 °C with shaking at 120 rpm for seven days. Oxygen saturation was monitored over time. A positive control containing 0.2 g l⁻¹ YE and 0.5 g l^− 1^ glucose was included for comparison. Abiotic controls supplemented with potassium, Casamino Acids, the mixture of 6 vitamins, and glucose, but without inoculum (CC) were used to account for non-biological oxygen consumption. Each experimental condition was tested at least in triplicate, and data are presented as mean values with standard deviations.

No NaCl was added to these respiration assays.

### Evaluation of amino acid requirements for respiratory activity

To investigate which amino acids may be essential for sustaining the respiratory activity of *Sb. thermosulfidooxidans*, a series of omission experiments was conducted. L-amino acids were clustered into six groups based on their biosynthetic origin and structural characteristics. The following groupings and final concentrations were used, reflecting a twofold increase relative to the content of 0.15 g l⁻¹ CA (casein hydrolysate, Carl Roth 2021): the glutamate family (45.30 µg ml⁻¹ Glu, 24.60 µg ml⁻¹ Pro, 8.40 µg ml⁻¹ Arg), the serine family (12.00 µg ml⁻¹ Ser, 4.80 µg ml⁻¹ Gly, 0.06 µg ml⁻¹ Cys), the aspartate family (16.50 µg ml⁻¹ Asp, 5.10 µg ml⁻¹ Met, 9.60 µg ml⁻¹ Thr, 19.80 µg ml⁻¹ Lys), the group containing alanine and branched-chain amino acids (7.80 µg ml⁻¹ Ala, 14.10 µg ml⁻¹ Val, 18.60 µg ml⁻¹ Leu, 10.50 µg ml⁻¹ Ile), the aromatic amino acids (7.50 µg ml⁻¹ Tyr, 7.80 µg ml⁻¹ Phe), and histidine (6.31 µg ml⁻¹ His) considered individually.

Asparagine and glutamine were not supplemented separately, as they undergo deamidation during acid hydrolysis and thus contribute to the pools of Asp and Glu, respectively (Fountoulakis and Lahm 1998). Tryptophan was initially excluded based on supplier documentation, but its role was evaluated separately due to its potential presence in CA and YE, as suggested by previous studies (Nagappa et al. 2022). No measurable influence on respiratory activity was observed under the tested conditions (data not shown).

Each test medium lacked one entire amino acid group, allowing functional inferences by comparison. All media were prepared in modified iron-free Mackintosh (1978) solution supplemented with 220 mg l^-1^ CoCl_2_*6 H_2_O and 0.5 g l⁻¹ glucose. A positive control with 0.2 g l⁻¹ YE and a chemical control without bacterial inoculum were included in all assays. Cultures were incubated at 45 °C and 120 rpm for seven days. Oxygen saturation was monitored daily during the first four days and again on day seven, as described in Sect. 2.3. No NaCl was added to these assays.

### Molecular weight fractionation of YE by ultrafiltration and nanofiltration

To identify the molecular weight range of active components in YE relevant to *Sb. thermosulfidooxidans* metabolism, stepwise membrane filtration of a 50 mg ml⁻¹ YE solution (BioChemica, AppliChem) was performed. Ultrafiltration was carried out using centrifugal filter devices with nominal molecular weight cut-offs (MWCO) of 3 kDa (Amicon Ultra-15, PLBC Ultracel-3, Millipore), and 1 kDa (Microsep Advance, 1 K MWCO). Filtration conditions followed the manufacturer’s instructions regarding centrifugation speed. Each filtration step was continued until the complete sample volume had passed through the membrane, resulting in filtrates of approximately 10 ml per condition.

The resulting filtrate fractions were incorporated into modified iron-free Mackintosh medium (pH 1.8) with 220 mg l^-1^ CoCl_2_*6 H_2_O at a volume equivalent to 0.2 g l⁻¹ of the original YE preparation, serving as the positive control. The medium was supplemented with 0.5 g l⁻¹ glucose. Cultures were incubated at 45 °C with shaking at 120 rpm for seven days. Oxygen saturation was measured at multiple timepoints (day 1, 2, 3, 4, and 7) using the procedure described in Sect. 2.3.

For higher resolution fractionation, nanofiltration was conducted using the Microdyn Nadir™ NP030 membrane with a molecular weight cut-off of 500 Da and an active filtration area of 0.0028 m². A total of 250 ml of YE solution (pre-filtered through the 3 kDa membrane) served as feedstock for nanofiltration. The process was conducted at room temperature (approximately 21 °C) and 20 bar of transmembrane pressure, until 50% of the feed volume had permeated. System performance was verified via pre- and post-rinsing with deionized water to evaluate fouling and membrane scaling.

The resulting filtrate (< 500 Da) and retentate (enriched in compounds with molecular weights above the nominal 500 Da cut-off) fractions were then tested in respiration assays. Each fraction was added in the same volume as used for 0.2 g l⁻¹ YE in the control condition. All test media contained the same supplement mixture as above, including glucose. Oxygen consumption was monitored on days 1 through 4 and again on day 7.

These fractionation experiments were performed using YE as the starting material and were independent of the subsequent FT-ICR-MS analyses performed on CA-derived material.

### Molecular weight fractionation of CA by ultrafiltration

To evaluate the size range of bioactive components present in CA (Carl Roth), a similar ultrafiltration approach was employed as described for YE (see Sect. 2.6). A 50 mg ml⁻¹ solution of CA (Carl Roth, casein hydrolysate, 2021) was subjected to sequential membrane filtration using centrifugal devices with MWCOs of 3 kDa (Amicon Ultra-15, PLBC Ultracel-3, Millipore) and 1 kDa (Microsep Advance). All filtration steps were conducted under the conditions recommended by the manufacturer and continued until the full sample volume had passed through the membrane, yielding filtrates of approximately 10 ml per step.

The collected CA filtrate fractions were added to iron-free modified Mackintosh (1978) medium (pH 1.8) supplemented with 220 mg l^-1^ CoCl_2_*6 H_2_O and 0.5 g l⁻¹ glucose and 0.03 g l⁻¹ potassium sulfate in a volume corresponding to 0.6 g l⁻¹ of the original CA solution. Cultures of *Sb. thermosulfidooxidans* were inoculated with 10% (v/v) preculture and incubated at 45 °C and 120 rpm for seven days. Respiratory activity was assessed via oxygen saturation measurements performed on days 1 through 3 and again on day 7, following the method outlined in Sect. 2.3. No NaCl was added to these assays.

### Characterization of CA-derived metabolites by ESI-FT-ICR-MS after ENV⁺ solid-phase extraction

High-resolution mass spectrometry was used to characterize cultivation-associated changes in low-molecular-weight compounds originating from CA. Importantly, the FT-ICR-MS experiments described in this section were performed with CA and CA-derived material, not with YE fractions.

*Sb. thermosulfidooxidans* cultures were grown in modified Mackintosh (1978) medium (pH 1.8) supplemented with 220 mg l^− 1^ CoCl_2_*6 H_2_O and 0.5 g l⁻¹ glucose, 0.03 g l⁻¹ potassium sulfate, and either 0.6 g l⁻¹ CA or an equivalent volume of CA filtrate previously processed via 1 kDa ultrafiltration. Inocula were added at 10% (v/v) from precultures, and flasks were incubated at 45 °C with shaking at 120 rpm. Bottles were sealed with rubber stoppers to allow sterile sampling using syringes. Culture supernatants were collected at the beginning of incubation (t0) and after seven days (t7) and were immediately frozen at − 80 °C until extraction. Corresponding chemical controls containing the same medium components but no bacterial inoculum were sampled at the same time points.

Metabolite extraction was performed using Isolute ENV+ solid-phase extraction columns as described previously (Kwiecień et al. 2025). The solid-phase resin was first activated by rinsing twice with 200 µl of 50% (v/v) acetonitrile in water, followed by equilibration using two 200 µl volumes of a 5% (v/v) acetonitrile solution containing 0.5% trifluoroacetic acid (TFA). Each sample was pretreated with 50 µl of this TFA–acetonitrile solution to achieve final concentrations of 0.5% TFA and 5% acetonitrile before being applied in duplicate (2 × 200 µl) to the column. Following binding, columns were washed twice with 200 µl of the same 0.5% TFA in 5% acetonitrile solution. Elution was performed using 10 µl of 70% acetonitrile, and eluates were dried under vacuum. A minimum of 1 mg of dried extract was recovered for downstream analysis.

Dried samples were reconstituted in 1 ml of ultrapure water using ultrasonic treatment (1 min) and mixed in a 1:1 ratio with methanol (LiChrosolv^®^, purity ≥ 99.8%, Merck Supelco). Solutions were filtered using 0.2 μm PTFE syringe filters (VWR) and immediately subjected to mass spectrometric analysis using electrospray ionization Fourier-transform ion cyclotron resonance mass spectrometry (ESI-FT-ICR-MS). A solvent mixture of 500 µl distilled water and 500 µl methanol served as a procedural blank. Each sample and blank was analyzed in both positive [ESI(+)] and negative [ESI(–)] ionization modes. Instrument parameters were as follows: capillary voltage + 3,400 V (ESI–) and − 3,400 V (ESI+), end plate offset − 500 V, nebulizer pressure 1.0 bar, dry gas 4.0 l min⁻¹ at 300 °C, syringe pump flow rate 10 µl min⁻¹. Mass spectra were recorded over an m/z range of 153.48–5000.00 Da, with 256 scans accumulated per run and resolution set to 2 million.

Data preprocessing, peak detection, molecular formula assignment, blank correction, data filtering, and visualization were performed using the Bruker Data Analysis 5.0 (SR1) and custom routines in MATLAB R2024a, following previously published protocols (Sander et al. 2023; Zuber et al. 2017).

Molecular formulas retained after filtering were further classified using van Krevelen diagrams based on their calculated H/C and O/C ratios (Rivas-Ubach et al. 2018). Six broad compositional regions were distinguished: lipid-like, peptide/protein-like, amino-sugar-like, carbohydrate-like, condensed-hydrocarbon-like, and lignin-like formulas. Van Krevelen distributions were evaluated separately for ESI(−) and ESI(+) datasets and compared between samples collected at the beginning of incubation (t0) and after seven days (t7).

### In-silico screening for peptide-compatible molecular formulas

An in-silico library of all possible di- and tripeptide compositions was generated computationally from the elemental formulas of the 20 standard proteinogenic amino acids. Peptide elemental formulas were calculated by summing the elemental compositions of the constituent amino acids and subtracting one H₂O molecule for each peptide bond. Exact neutral monoisotopic masses were calculated from the resulting C, H, N, O, and S compositions. The corresponding theoretical ions were calculated as [M − H]^−^ for negative-ion mode and [M + H]^+^ for positive-ion mode.

Molecular formulas assigned to FT-ICR-MS signals were screened separately for ESI(−) and ESI(+) datasets by exact matching of their C, H, N, O, and S elemental compositions to entries in the in-silico peptide library. Matching was performed using the complete elemental formula rather than rounded *m/z* values. For formula-matched signals, the experimentally observed *m/z* was additionally checked for consistency with the theoretical [M − H]^−^ or [M + H]^+^*m/z* values. Peptide candidates sharing the same elemental formula were collapsed into a single molecular-formula group, and all compatible amino acid compositions were retained. Consequently, assignments were reported as candidate peptide compositions rather than unequivocally identified peptide sequences.

Peptide-compatible molecular formulas were compared between samples collected at the beginning of incubation (t0) and after 7 days (t7). The same analysis was independently performed for the corresponding uninoculated chemical controls (CC). The detection status was assessed separately for each ionization mode. A signal was classified as showing culture-associated disappearance when it was detected in the inoculated culture at t0, was no longer detected at t7, and remained detectable in the corresponding uninoculated chemical control at both t0 and t7. Signals that disappeared concurrently from the chemical control were not considered culture-associated. Relative ESI signal intensities were treated as semi-quantitative and were used for descriptive evaluation of temporal signal changes.

For Table 1, only peptide-compatible signals classified as showing culture-associated disappearance were included, i.e. signals detected in the inoculated culture at t0, no longer detected at t7, and retained in the corresponding uninoculated chemical control at both time points. This resulted in 28 ionization-mode-specific observations corresponding to 26 unique molecular formulas. The complete set of peptide-compatible signals detected in inoculated cultures at t0 and/or t7 is provided in Online Resource Table S1. Identical elemental formulas qualifying in both ionization modes were retained as separate mode-specific observations in Table 1 because their relative ESI intensities were evaluated independently.

**Table 1:** Peptide-compatible molecular formulas showing loss of detectability in inoculated cultures while remaining detectable in uninoculated chemical controls. 28 ionization-mode-specific observations corresponding to 26 unique molecular formulas

Molecular formula	Candidate peptide composition(s)	Calculated neutral monoisotopic mass (Da)	ESI mode	Culture t0 relative ESI intensity (base peak = 100)	CC t0 relative ESI intensity (base peak = 100)	CC t7 relative ESI intensity (base peak = 100)
C9H18N2O4	Ser + Ile; Ser + Leu; Val + Thr	218.126657	ESI(+)	0.2917	0.4984	0.1409
C9H18N2O4	Ser + Ile; Ser + Leu; Val + Thr	218.126657	ESI(−)	0.1004	0.1196	0.2420
C10H20N2O4	Ile + Thr; Leu + Thr	232.142307	ESI(−)	0.1547	0.1383	0.3503
C12H16N2O3	Ala + Phe	236.116092	ESI(+)	0.0629	0.1266	0.0937
C11H23N3O3	Val + Lys	245.173942	ESI(+)	0.4483	0.7186	0.3368
C10H18N2O3S	Met + Pro	246.103814	ESI(+)	0.1948	0.3897	0.0804
C10H18N2O5	Ile + Asp; Leu + Asp; Val + Glu	246.121572	ESI(−)	0.3306	0.5693	0.2889
C12H16N2O4	Ala + Tyr; Ser + Phe	252.111007	ESI(+)	0.2005	0.4181	0.1462
C11H18N4O3	Val + His	254.137890	ESI(+)	0.0811	0.1187	0.0709
C13H23N3O4	Ala + Val+Pro; Gly + Ile+Pro; Gly + Leu+Pro	285.168856	ESI(+)	0.1971	0.4414	0.1726
C14H18N2O5	Phe + Glu	294.121572	ESI(+)	0.1196	0.3271	0.1061
C13H25N3O5	Ala + Ile+Thr; Ala + Leu+Thr; Ser + Val+Val	303.179421	ESI(+)	0.0984	0.1720	0.1123
C14H18N2O6	Tyr + Glu	310.116486	ESI(+)	0.1339	0.4777	0.1177
C14H20N2O4S	Met + Tyr	312.114378	ESI(+)	0.0565	0.2107	0.0720
C18H20N2O3	Phe + Phe	312.147393	ESI(+)	0.2306	0.4423	0.2230
C14H25N3O5	Ser + Ile+Pro; Ser + Leu+Pro; Val + Thr+Pro	315.179421	ESI(+)	0.5827	1.2936	0.4636
C18H20N2O4	Phe + Tyr	328.142307	ESI(+)	1.1830	2.2639	1.0026
C13H25N3O5S	Cys + Ile+Thr; Cys + Leu+Thr; Ser + Val+Met	335.151492	ESI(+)	0.0563	0.1324	0.1117
C15H25N3O6	Ile + Asp+Pro; Leu + Asp+Pro; Val + Glu+Pro	343.174336	ESI(+)	0.2081	0.4796	0.1395
C15H27N3O6	Val + Ile+Asp; Val + Leu+Asp; Val + Val+Glu	345.189986	ESI(−)	0.0714	0.1276	0.0964
C15H30N4O5	Ser + Ile+Lys; Ser + Leu+Lys; Val + Thr+Lys	346.221620	ESI(+)	0.0976	0.1334	0.0972
C12H23N3O5S2	Cys + Thr+Met	353.107913	ESI(+)	0.0915	0.1851	0.0611
C16H27N3O6	Ile + Glu+Pro; Leu + Glu+Pro	357.189986	ESI(+)	0.3012	0.7984	0.1840
C16H27N3O6	Ile + Glu+Pro; Leu + Glu+Pro	357.189986	ESI(−)	0.2101	0.6082	0.3076
C17H31N3O6	Ile + Ile+Glu; Leu + Ile+Glu; Leu + Leu+Glu	373.221286	ESI(+)	0.1572	0.4093	0.1156
C20H29N3O4	Ile + Phe+Pro; Leu + Phe+Pro	375.215806	ESI(+)	0.1137	0.2387	0.1016
C17H27N5O6	Ile + Glu+His; Leu + Glu+His	397.196134	ESI(+)	0.2010	0.5529	0.2214
C18H36N6O4	Ile + Ile+Arg; Leu + Ile+Arg; Leu + Leu+Arg	400.279804	ESI(+)	0.3669	0.4550	0.3652

Note. All listed signals were detected in the inoculated culture at t0, were not detected at t7 under the applied analytical conditions, and remained detectable in the corresponding uninoculated chemical control (CC) at both t0 and t7. Relative ESI signal intensities were normalized to the base-peak intensity of the respective spectrum (base peak = 100) and are considered semi-quantitative. Candidate peptide compositions represent amino acid compositions compatible with the assigned elemental formula and exact mass. Sequence order cannot be resolved by this approach, and leucine and isoleucine cannot be distinguished

Computational analyses were performed using Python v3.12.3 with pandas v2.3.2 and openpyxl v3.1.5.

## Results

### Determination of chloride tolerance

The comparative assessment of chloride tolerance among the selected *Sulfobacillus* species revealed pronounced differences in chloride tolerance under the tested assay conditions (Online Resource 1). Among the tested type strains, *Sb. thermosulfidooxidans* DSM 9293^T^ showed the highest tolerance, with biological Fe(II) oxidation remaining detectable up to 500 mM NaCl, corresponding to approximately 17.7 g Cl⁻ l⁻¹. The lowest tolerance was observed for *Sb. acidophilus* DSM 10,332^T^, with biological Fe(II) oxidation remaining detectable up to 50 mM NaCl (1.77 g Cl⁻ l⁻¹ ). The highest NaCl concentrations at which biological Fe(II) oxidation remained detectable were 75 mM (2.66 g Cl⁻ l⁻¹) for *Sb. sibiricus* DSM 17363T, 125 mM (4.43 g Cl⁻ l⁻¹) for *Sb. harzensis* DSM 109850T, and 175 mM (6.20 g Cl⁻ l⁻¹) for *Sb. benefaciens* DSM 19468T.

### Assessment of the combined effect of CA, vitamins, and potassium on respiratory activity

**Fig. 1:**
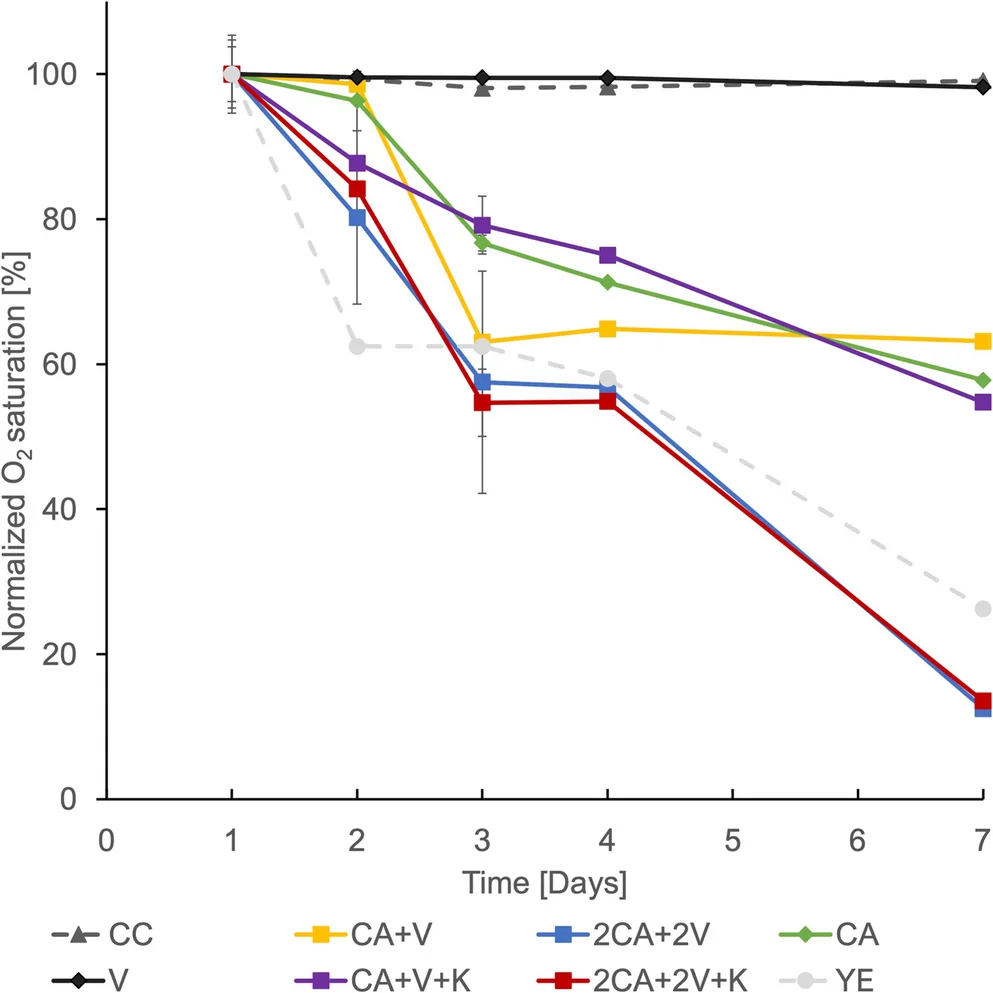
Respiratory response of *Sb. thermosulfidooxidans* over 7 days of incubation with glucose and various additional supplements (potassium, casamino acids, or a mixture of 6 vitamins). The tested conditions included: K − 0.03 g l⁻¹ K₂SO₄, CA − 0.3 g l⁻¹ Casamino acids, 2CA–0.6 g l⁻¹ CA, V - vitamin mixture, 2 V – doubled concentrated vitamin mixture, YE − 0.2 g l^− 1^ yeast extract, CC – chemical control supplemented with potassium, casamino acids, the mixture of 6 vitamins, and glucose (without bacteria). O₂ saturation values were normalized to the first stabilized measurement (t1 = 100%). Lower normalized O₂ saturation remaining indicates greater oxygen consumption and respiratory activity. Data are presented as mean ± SD of *n* = 3 independent replicates

Supplement composition strongly influenced the respiratory response of *Sb. thermosulfidooxidans* during the seven-day incubation period (Fig. 1; Online Resource 2). Abiotic controls remained close to the normalized starting O₂ saturation, confirming that the decreases observed in inoculated cultures reflected predominantly biological oxygen consumption.

In the experiment shown in Fig. 1, the strongest oxygen depletion occurred in cultures supplemented with doubled concentrations of CA and vitamins with or without additional potassium (2CA+2 V, 2CA + 2 V + K). In both treatments, the remaining O₂ saturation decreased to approximately 13% by day 7, compared with approximately 26% in the YE control. In contrast, vitamins alone produced essentially no detectable respiratory stimulation, with O₂ saturation remaining close to 100% throughout the experiment. Treatments containing CA at the lower concentration showed intermediate responses, reaching approximately 55–63% remaining O₂ saturation by day 7.

The additional experiment presented in Online Resource 2 confirmed the limited effect of vitamins alone and the substantially greater respiratory response associated with CA-containing supplementation. Here, the YE control reached approximately 46% O₂ saturation remaining by day 7, whereas CA-containing treatments reached approximately 54–59%. Thus, although the magnitude of the response varied between experimental series, CA-containing supplementation consistently supported substantial glucose-dependent respiration, whereas vitamins alone had little effect.

### Evaluation of amino acid requirements for respiratory activity

**Fig. 2:**
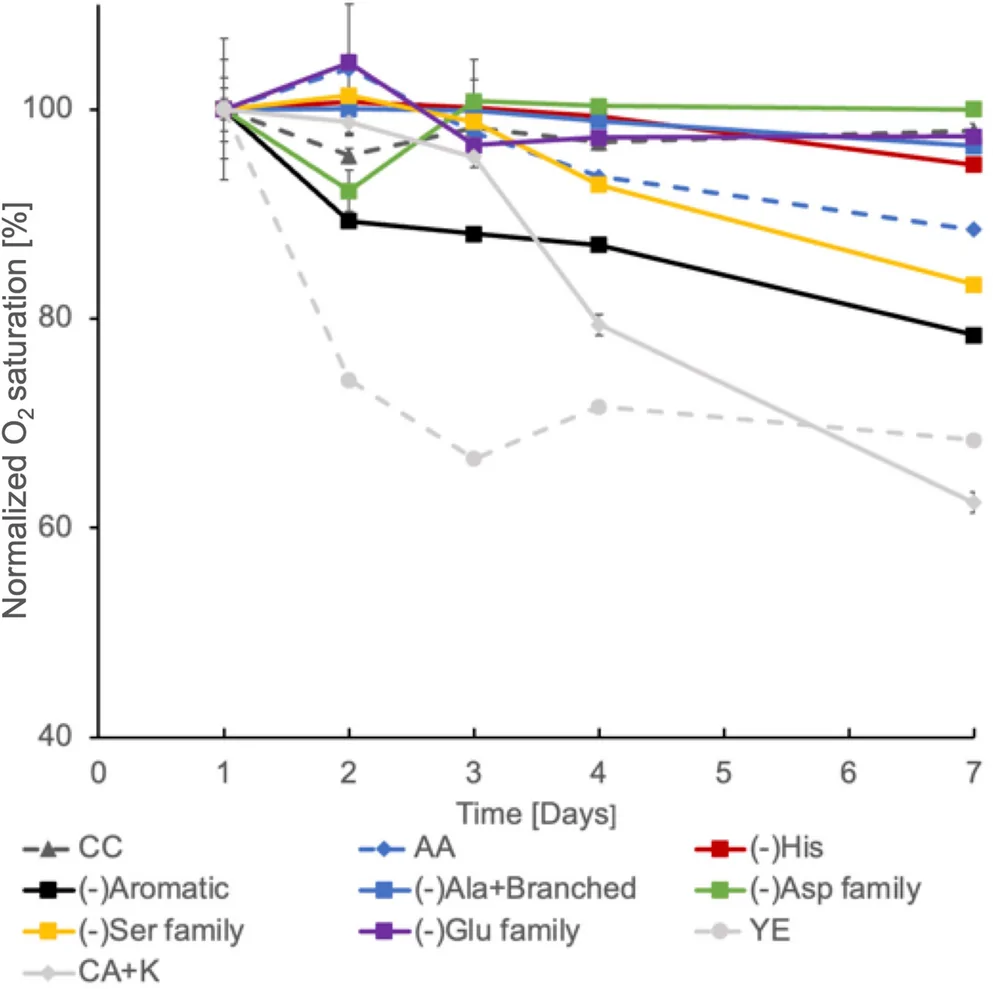
Assessment of the role of selected amino acid groups in supporting the respiratory activity of *Sb. thermosulfidooxidans* in the presence of glucose. Shown is the 7-day time course of O₂ saturation for cultures supplemented with 0.2 g l⁻¹ yeast extract as a positive control (YE), a complete amino acid mixture (AA), with omission of the indicated amino acid group (-), a mixture of casamino acid with potassium (CA + K), and an abiotic chemical control (CC) supplemented with complete amino acid mixture, potassium, and glucose. O₂ saturation values were normalized to the first stabilized measurement (t1 = 100%); lower values indicate greater oxygen consumption and respiratory activity. Data are presented as mean ± SD of *n* = 3 independent replicates

The complete amino acid mixture (AA) supported only limited respiratory activity, with normalized O₂ saturation remaining at approximately 89% after seven days (Fig. 2). In contrast, YE caused a substantially stronger decrease in O₂ saturation, reaching approximately 68% by day 7.

Omission of individual amino acid groups revealed marked differences in their contribution to respiration. Removal of the glutamate family, the alanine/branched-chain amino acid group, histidine, or the aspartate family strongly reduced oxygen depletion relative to the complete AA mixture. At day 7, cultures lacking these groups retained approximately 94–100% of the initial O₂ saturation, close to the uninoculated chemical control (~ 97%). These results indicate that the presence of these amino acid groups contributed to the limited respiratory activity supported by the complete amino acid mixture.

In contrast, omission of the serine family or aromatic amino acids resulted in greater oxygen depletion than observed with the complete AA mixture, with approximately 82% and 78% O₂ saturation remaining, respectively, at day 7. This reproducible but unexpected response suggests that omission of these groups did not impair respiratory activity under the tested conditions; its physiological basis remains unclear.

The CA + K treatment showed a distinct temporal response compared with YE. YE promoted a more rapid decrease in O₂ saturation during the first days of incubation, whereas the response to CA + K was delayed. Nevertheless, by day 7, the CA + K treatment reached approximately 62% O₂ saturation remaining, compared with approximately 68% for YE. Thus, despite its slower onset, by the end of the assay CA + K supported oxygen depletion comparable to, and slightly greater than, that observed with YE.

### Molecular weight fractionation of YE by ultrafiltration and nanofiltration

**Fig. 3:**
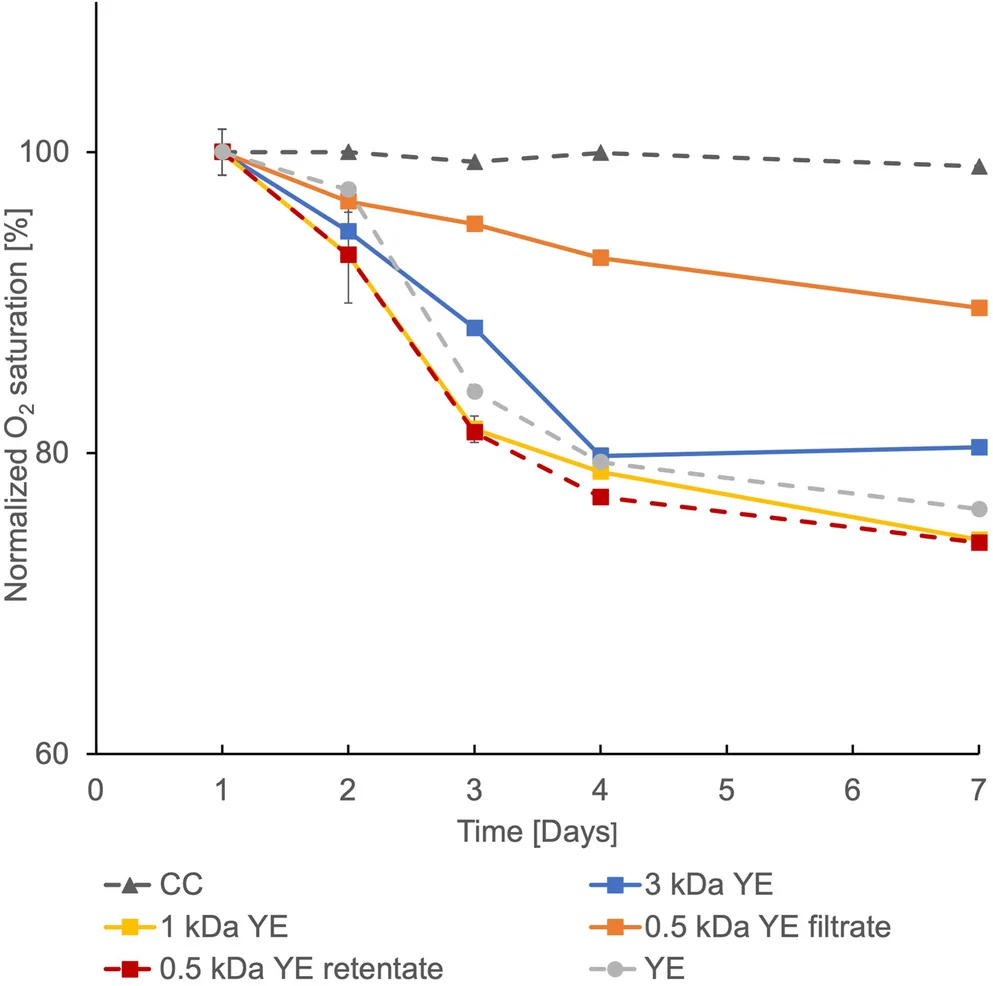
Effect of molecular-weight fractionation of yeast extract on glucose-dependent respiration of *Sb. thermosulfidooxidans*. YE was fractionated using membranes with nominal molecular-weight cut-offs (MWCOs) of 3 kDa, 1 kDa, and 0.5 kDa. The < 3-kDa and < 1-kDa fractions represent membrane permeates, whereas the 0.5-kDa permeate and corresponding > 0.5-kDa-enriched retentate were tested separately. Unfractionated YE was included as a positive control, and CC represents an uninoculated chemical control. O₂ saturation values were normalized to the first stabilized measurement (t1 = 100%). Lower remaining O₂ saturation indicates greater respiratory activity. Data are presented as mean ± SD of *n* = 3 independent replicates

Molecular-weight fractionation of YE revealed clear differences in the ability of the resulting fractions to support glucose-dependent respiration of *Sb. thermosulfidooxidans* (Fig. 3). The uninoculated chemical control remained close to the normalized starting O₂ saturation throughout the experiment.

The < 500-Da YE permeate produced only a limited decrease in O₂ saturation, with approximately 89% O₂ saturation remaining after seven days. In contrast, the corresponding > 500-Da-enriched retentate supported substantially greater oxygen depletion, reaching approximately 74–75% O₂ saturation remaining by day 7, comparable to unfractionated YE (~ 76%).

Material passing the nominal 1-kDa membrane also retained strong respiration-stimulating activity, resulting in approximately 74–75% O₂ saturation remaining after seven days. The < 3-kDa fraction showed an intermediate response, with approximately 80% O₂ saturation remaining. Taken together, these results indicate that a substantial proportion of the respiration-stimulating activity of YE was associated with compounds retained above the nominal 500-Da cut-off while remaining sufficiently small to pass a nominal 1-kDa membrane. Thus, the active material was enriched within the approximate 0.5–1-kDa molecular-weight range.

### Molecular weight fractionation of CA by ultrafiltration

**Fig. 4:**
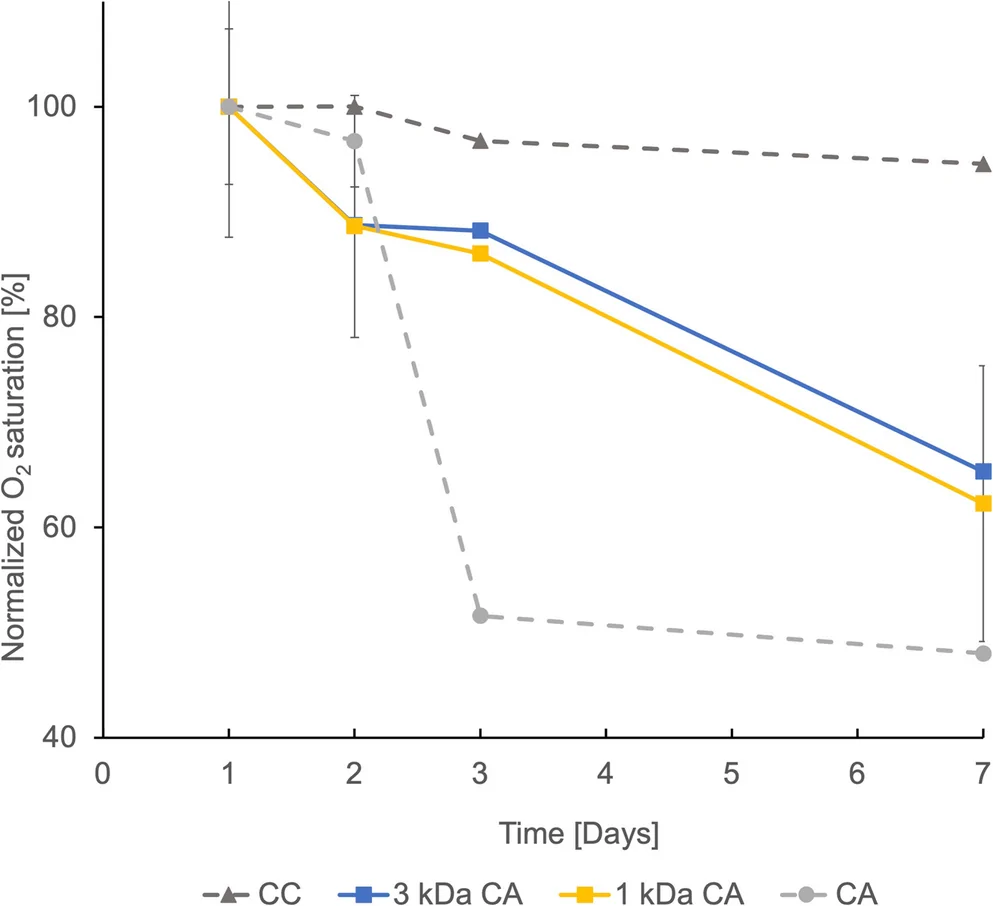
Effect of molecular-weight fractionation of casamino acids on glucose-dependent respiration of *Sb. thermosulfidooxidans*. Unfractionated casamino acids (CA) and CA permeates obtained using membranes with nominal molecular-weight cut-offs of 3 kDa and 1 kDa were tested in iron-free modified Mackintosh medium supplemented with glucose and potassium. CC represents an uninoculated chemical control. O₂ saturation values were normalized to the first stabilized measurement (t1 = 100%); lower remaining O₂ saturation indicates greater respiratory activity. Data are presented as mean ± SD of *n* = 3 independent replicates

Fractionation of CA substantially reduced its ability to support glucose-dependent respiration of *Sb. thermosulfidooxidans* compared with unfractionated CA (Fig. 4). After seven days, cultures supplemented with unfractionated CA reached approximately 48% normalized remaining O₂ saturation, whereas the < 1-kDa and < 3-kDa CA fractions retained approximately 62% and 65% O₂ saturation, respectively. The uninoculated chemical control remained close to the initial O₂ saturation throughout the experiment (~ 94% at day 7).

The < 1-kDa and < 3-kDa fractions produced similar respiratory responses, indicating that further restriction of the permeate from < 3 kDa to < 1 kDa did not markedly alter its respiration-supporting capacity. However, both fractions were less effective than unfractionated CA. These results suggest that the full respiratory effect of CA cannot be attributed exclusively to compounds below 1 or 3 kDa and may depend on additional higher-molecular-weight constituents and/or interactions among multiple components of the unfractionated hydrolysate.

### FT-ICR-MS characterization of cultivation-associated changes in CA-derived compounds

**Fig. 5:**
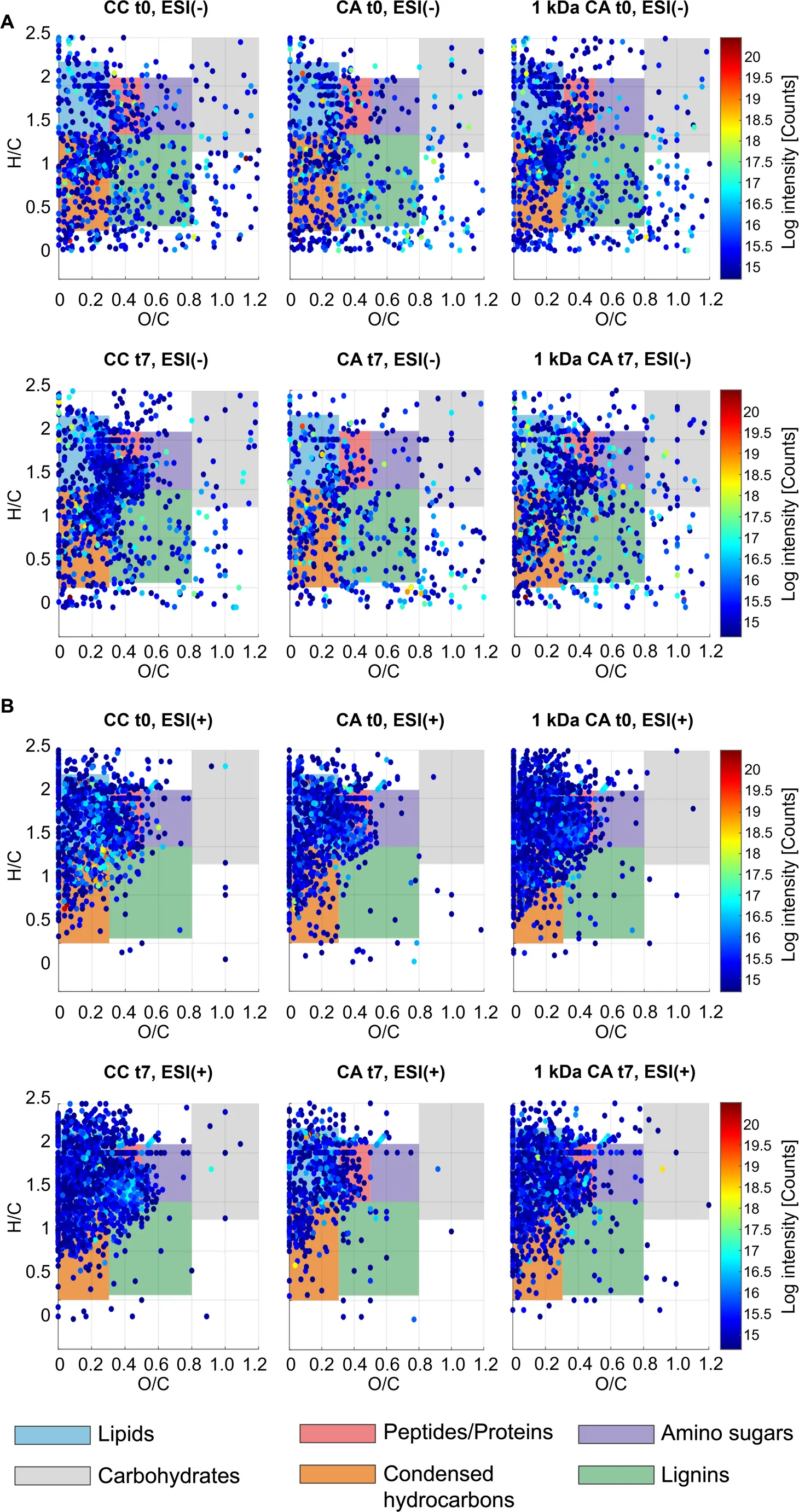
Van Krevelen diagrams derived from ESI(-)-FT-ICR-MS (**A**) and ESI(+)-FT-ICR-MS (**B**) analyses of culture supernatants collected at the beginning of the incubation (t0) and after 7 days (t7). Samples originated from *Sb. thermosulfidooxidans* cultures grown in modified Mackintosh (1978) medium (pH 1.8) supplemented with glucose, and either casamino acids (CA) or a 1 kDa ultrafiltrated fraction of casamino acids (1 kDa CA). Metabolites were extracted using ENV⁺ solid-phase extraction columns. Chemical controls (CC) contained medium and CA without bacterial inoculation. Signal classification was performed according to the criteria of Rivas-Ubach et al. (2018). Signal intensities are represented on a logarithmic scale using a color gradient: blue for low, yellow-green for intermediate, and red for high intensity levels (see accompanying color scale). These analyses were performed on CA-derived samples and are independent of the YE molecular-weight fractionation shown in Fig. 3

FT-ICR-MS analysis revealed a chemically diverse pool of low-molecular-weight compounds in the culture media containing unfractionated CA or the < 1-kDa CA fraction (Fig. 5). Van Krevelen diagrams were generated separately for negative- and positive-ion electrospray ionization modes (Fig. 5A and B, respectively) and compared between the beginning of incubation (t0) and day 7 (t7).

In ESI(−) mode (Fig. 5A), molecular formulas were broadly distributed across regions commonly associated with lipid-like, peptide/protein-like, amino-sugar-like, carbohydrate-like, lignin-like, and condensed-hydrocarbon-like compositions. The overall distributions remained relatively broad after seven days, although changes in signal density and signal intensity were apparent in both CA-containing samples and the uninoculated chemical control.

In ESI(+) mode (Fig. 5B), the detected molecular formulas were concentrated predominantly within the peptide/protein-like, lipid-like, amino-sugar-like, and condensed-hydrocarbon-like regions. Both unfractionated CA and the < 1-kDa CA fraction showed changes in the number and distribution of detected features between t0 and t7. However, temporal changes were also observed in the uninoculated control (CC, graphs on the left). Therefore, differences in signal number or intensity were interpreted conservatively as changes in sample composition during incubation rather than as direct evidence of biological uptake.

The FT-ICR-MS analysis was subsequently used to identify molecular formulas compatible with low-molecular-weight peptide compositions in the CA-derived samples. These assignments were evaluated separately from the molecular-weight fractionation of YE.

Screening against the in-silico di- and tripeptide library revealed a broad diversity of peptide-compatible molecular formulas in the CA-derived cultivation samples. In ESI(−) mode, 44 peptide-compatible formulas were detected at t0 and 56 at t7, representing 65 different formulas across both time points. In ESI(+) mode, 101 formulas were detected at t0 and 61 at t7, representing 109 different formulas across both time points. After accounting for formulas occurring at multiple time points and/or in both ionization modes, a total of 118 unique elemental formulas compatible with di- or tripeptide compositions were detected in the inoculated cultures (Online Resource Table S1). Of these, 56 were detected in both ESI(−) and ESI(+) modes. Several formulas were compatible with more than one amino acid composition, particularly where isomeric residues such as Leu and Ile were involved. They were therefore reported as candidate peptide compositions.

Of the 118 unique formulas, 107 were detected at t0 and 82 at t7. Eleven formulas were detected only at t7, whereas 36 were detected only at t0. The remaining 71 formulas were detected at both time points. Signals detected only at t7 may represent compounds formed or released during incubation, for example through transformation or hydrolysis of larger peptide constituents. Their origin could not be resolved from the present data and was therefore not interpreted further.

Among the peptide-compatible signals detected at t0, 57 mode-specific signals were no longer detected in the inoculated cultures after seven days. Comparison with the corresponding uninoculated chemical controls showed that 28 of these signals, representing 26 unique molecular formulas, remained detectable in the controls at both t0 and t7 and were therefore classified as showing culture-associated disappearance (Table 1). Eighteen additional signals disappeared from both the inoculated cultures and the chemical controls, whereas for 11 signals the control comparison was incomplete or ambiguous.

Two molecular formulas, C_9_H_18_N_2_O_4_ and C_16_H_27_N_3_O_6_, showed culture-associated disappearance in both ionization modes. C_9_H_18_N_2_O_4_ was compatible with the dipeptide compositions Ser/Ile, Ser/Leu, or Val/Thr, whereas C_16_H_27_N_3_O_6_ was compatible with the tripeptide compositions Ile/Glu/Pro or Leu/Glu/Pro. Because elemental formula and accurate mass do not resolve residue order or structural isomers, these signals were not assigned to individual peptide sequences. Their culture-specific disappearance is consistent with microbial utilization or transformation of the corresponding compounds, although the present analysis does not resolve their exact biochemical fate.

## Discussion

The present study aimed to systematically assess chloride tolerance among several *Sulfobacillus* species, to identify strains possibly suitable for bioleaching under saline conditions, and to elucidate molecular factors supporting metabolism of the halotolerant *Sb. thermosulfidooxidans*. The comparative evaluation of chloride tolerance across five *Sulfobacillus* type strains clearly demonstrates pronounced physiological diversity within this genus, particularly regarding their ability to withstand osmotic and ionic stress under acidic conditions. Among the tested species, *Sb. thermosulfidooxidans* exhibited a notable halotolerance, maintaining ferrous iron oxidation activity at NaCl concentrations up to 500 mM (approximately 17.7 g Cl^-^ l^-1^), a finding consistent with earlier studies reporting its sustained activity under prolonged exposure to saline conditions, and supporting the feasibility of its application in seawater-based bioleaching systems (Huynh et al. 2020a; b). However, in previous studies (Huynh et al. 2019), *Sb. thermosulfidooxidans* demonstrated mineral-specific responses during bioleaching, showing no inhibition at 200 mM NaCl in chalcopyrite leaching, while its activity was limited with sphalerite at an identical chloride concentration. Thus, for a possible use of seawater, a dependence of the chloride tolerance on the mineral substrate or an increased jarosite formation resulting from sodium ion availability may additionally have to be considered (Huynh et al. 2019). The comparative chloride-tolerance values should be interpreted as condition-specific rather than as absolute species-specific maxima. The reported temperature optima of the tested species differ considerably, ranging from 38.5 °C for *Sb. benefaciens* to 55 °C for *Sb. sibiricus*, while optima of 45 °C, 45–50 °C, and 50–55 °C have been reported for *Sb. harzensis*, *Sb. acidophilus*, and *Sb. thermosulfidooxidans*, respectively (Zhang et al. 2021). Because the comparative assay was performed at 45 °C for four strains and at 37 °C for *Sb. benefaciens*, the apparent chloride-tolerance thresholds may partly reflect the assay temperature in addition to intrinsic species-specific tolerance.

Genomic and physiological investigations revealed that acidophili*c* strains employ distinct chloride tolerance strategies, including the upregulation of potassium transporters (Kdp, Ygg) and biosynthetic pathways for osmoprotectants such as ectoine and trehalose, with exogenous ectoine supplementation significantly enhancing minimum inhibitory concentrations (Rivera-Araya et al. 2020). Bobadilla-Fazzini et al. (2014) reported that *Sb. thermosulfidooxidans* strain Cutipay, despite harboring genes encoding 2-haloacid dehalogenase, exhibited complete growth inhibition at 8 g l⁻¹ (137 mM) NaCl and 21 g l⁻¹ (282 mM) KCl, highlighting strain-specific limits in halotolerance.

Additionally, substrate-dependent inhibition patterns were identified for *Sb. thermosulfidooxidans* DSM 9293^T^, with complete iron oxidation inhibition observed at 525 mM NaCl with ferrous sulfate, while higher tolerance thresholds were noted with tetrathionate (725 mM) and pyrite (800 mM) as substrates (Huynh et al. 2020a). Adaptation to saline conditions further conferred an increase in cellular respiration rates, with NaCl-adapted cells exhibiting up to a threefold increase in respiration at 200 mM NaCl relative to non-adapted cells (Huynh et al. 2020a).

An influence of pH on chloride tolerance has been recognized in several acidophilic microorganisms. Earlier work by Suzuki et al. (1999), performed with *Acidithiobacillus (Thiobacillus) thiooxidans*, demonstrated that the cells are more susceptible to chloride-induced stress at lower pH values, which was attributed to increased permeability of chloride ions under highly acidic conditions. Consistent with these observations, Falagán and Johnson (2018) showed that several acidophilic species, including strains of *Acidithiobacillus*, *Sulfobacillus*, and *Acidiphilium*, exhibited higher chloride tolerance at pH 3.0 compared to pH 2.0.

Comparative analyses of different microorganisms have demonstrated significant variability in their chloride tolerance and abilities to oxidize Fe²⁺ and sulfur during chalcopyrite bioleaching. *Sb. thermosulfidooxidans* exhibited relatively high chloride tolerance (31 g NaCl l⁻¹; 530 mM NaCl) (Huynh et al. 2019), maintaining iron oxidation activity and copper bioleaching capacity even at elevated chloride concentrations, in contrast to *Leptospirillum ferriphilum*, which experienced complete growth inhibition at 19 g NaCl l⁻¹ (325 mM NaCl) (Huynh et al. 2019). Although copper extraction by *Sb. thermosulfidooxidans* from chalcopyrite concentrate did not exceed 30%, it remained stable across all salinity conditions tested, indicating strong halotolerance (Huynh et al. 2019). In contrast, copper recovery by *L. ferriphilum* dropped to below 10% in the presence of 100 mM and 200 mM of NaCl (Huynh et al. 2019). Similarly, *At. ferrooxidans* was more sensitive to chloride during Fe²⁺ oxidation (with inhibition occurring around 200 mM chloride), while its sulfur oxidation activity remained largely unaffected even at concentrations up to 400 mM chloride (Harahuc et al. 2000). Thermophilic archaea, such as *Sulfolobus acidocaldarius*, have demonstrated the ability to sustain bioleaching activity in the presence of high chloride concentrations, with effective copper extraction observed at NaCl levels up to 58.5 g NaCl l⁻¹ (1 M NaCl) (Martins et al. 2019), while for *Acidianus manzaensis*, chloride tolerance under bioleaching conditions has been reported only at considerably lower concentrations (0.66 g NaCl l⁻¹; 11 mM NaCl) (Chang-Li et al. 2012).

The ability of *Sb. thermosulfidooxidans* to oxidize iron in the presence of 500 mM NaCl, although significant within *Sulfobacillus*, remains lower than that observed for some extremophilic halophiles such as *Alicyclobacillus* sp. SO9, which oxidized iron up to 1.5 M NaCl (~ 87 g NaCl l⁻¹) and strictly required NaCl for growth (Huynh et al. 2024). This obligate halophilic phenotype suggests a very efficient adaptation strategy, possibly involving membrane architecture, compatible solute accumulation, and ion transport systems specifically tuned for high salinity environments.

In the present study, the highest NaCl concentrations supporting detectable biological Fe(II) oxidation were 125 mM for *Sb. harzensis*, 75 mM for *Sb. sibiricus*, and 175 mM for *Sb. benefaciens*, illustrating a continuum of chloride tolerance phenotypes that may reflect species-specific adaptations to different environmental salinities. Based on its ability to sustain iron oxidation at the highest chloride concentrations among the tested species, *Sb. thermosulfidooxidans* DSM 9293^T^ was selected for deeper investigation as a promising candidate for bioleaching under high-salinity conditions, which could be pivotal for enhancing process efficiency in saline or seawater-based systems.

To systematically investigate the nutritional needs of *Sb. thermosulfidooxidans*, we employed an optical oxygen sensor system (PyroScience^®^) to monitor respiration activity at low pH (Kwiecień et al. 2025). While some variability in oxygen saturation levels was observed, potentially due to minor experimental limitations such as gas exchange inconsistencies, the approach allowed comparative assessment of metabolic responses to defined supplement compositions under controlled cultivation conditions.

Initial experiments conducted in glucose-based medium demonstrated that the respiratory activity of *Sb. thermosulfidooxidans* was highly responsive to organic and inorganic additives, indicating a strong dependence on external nutrient supplementation to sustain metabolic activity under the tested conditions. This observation is consistent with previous reports showing that yeast extract enhances the growth, iron oxidation, and chloride tolerance of *Sb. thermosulfidooxidans* (Huynh et al. 2020a). The addition of CA, with or without an accompanying vitamin mixture, consistently enhanced oxygen depletion, while the vitamin mixture alone had no effect and was obviously not the primary driver of metabolic stimulation (Fig. 1, Online Resource 2). This contrasts with findings for *Alicyclobacillus* sp. SO9, where thiamine and niacin were shown to be essential for activity (Kwiecień et al. 2025). Despite the apparent lack of a vitamin dependence, the nutritional requirements of *Sb. thermosulfidooxidans* remain complex, as evidenced by the current inability to establish a fully defined minimal medium. Moreover, doubling the concentration of CA resulted in further enhancement of respiratory activity, demonstrating a clear dose-dependent response (Fig. 1). In comparison to CA, the effect of potassium supplementation was less consistent and depended on the specific experimental conditions. While an increase in respiration was observed in the presence of potassium in Online Resource 2, particularly during the early incubation phase, this trend was less clearly resolved in Fig. 1. These observations suggest that potassium may contribute to respiratory activity, although its effect appears to reach saturation at relatively low concentrations. However, interpretation of potassium-specific effects is complicated by the potential presence of potassium in other medium components, such as casamino acids or the vitamin solution. Overall, the influence of potassium on respiration remained less pronounced and more variable than that of CA. Potassium may nevertheless contribute to ion balance and intracellular pH homeostasis in acidophilic microorganisms, as previously described under acidic and saline conditions (Falagán and Johnson 2018; Suzuki et al. 1999). However, because the nutrient-dissection experiments in the present study were performed without added NaCl, no conclusions can be drawn regarding a specific role of potassium in supporting respiration during chloride stress. To further dissect the organism’s amino acid dependencies, systematic omission experiments were performed under glucose-based, iron-free conditions. Removal of the glutamate family, aspartate family, histidine, or the alanine plus branched-chain amino acid group strongly reduced oxygen depletion, with final O₂ saturation values approaching those of the abiotic control (Fig. 2). These findings indicate that compounds belonging to these amino acid groups contribute substantially to the respiratory response under the tested conditions. However, the omission experiments do not establish an absolute requirement for growth, as respiratory activity rather than biomass formation or culture propagation was measured. Interestingly, the complete amino acid mixture (17 amino acids, excluding asparagine, glutamine, and tryptophan due to their expected hydrolysis (Fountoulakis and Lahm 1998) elicited only very low oxygen depletion (ca. 89% normalized O₂ saturation remaining), whereas YE consistently induced significantly higher respiration activity (ca. 68% remaining). This pattern suggests that the mixture of free amino acids may not provide the optimal composition or bioavailability required by *Sb. thermosulfidooxidans* for effective uptake and metabolism. A possible explanation for YE’s or CA’s superior performance lies in a potentially more efficient utilization of peptide-bound than free amino acids, as demonstrated in *Lactobacillus casei*, where peptides were shown to support more efficient growth than their constituent amino acids, likely due to more effective uptake and utilization (Kihara and Snell 1960). This interpretation is also compatible with genomic information available for *Sb. thermosulfidooxidans*. Comparative genome analysis of strain ST identified several amino acid and oligo-/dipeptide transport systems as well as leucyl- and methionyl-aminopeptidases, providing a potential molecular basis for the uptake and intracellular processing of peptide-bound amino acids (Guo et al. 2014). Nevertheless, the presence of these systems does not demonstrate preferential utilization of individual peptides under the conditions examined here. Overall, these findings are consistent with previous CHO cell culture studies showing that peptide-containing fractions of yeast extract can support cellular activity and that di- and tripeptides may serve as efficient nitrogenous substrates (Mosser et al. 2015).

To probe the molecular-size distribution of respiration-supporting components, YE and CA were subjected to membrane fractionation. For YE, the < 500-Da permeate supported only limited respiration, whereas the > 500-Da-enriched retentate and the < 1-kDa fraction retained activity comparable to unfractionated YE (Fig. 3). These results indicate that a substantial proportion of the respiration-supporting activity of YE was associated with compounds within an approximate nominal molecular-weight range of 0.5–1 kDa. This range should not, however, be interpreted as evidence for a specific compound class, particularly because membrane MWCO values represent operational rather than absolute molecular-size boundaries.

CA showed a different fractionation pattern. Unfractionated CA supported greater oxygen depletion than either the < 1-kDa or < 3-kDa fractions (Fig. 4), indicating that the low-molecular-weight fractions alone were insufficient to reproduce the full effect of the hydrolysate. This may reflect contributions from higher-molecular-weight constituents, losses during fractionation, or interactions among several CA components. Importantly, the YE fractionation and FT-ICR-MS analyses should be interpreted independently: the 0.5–1-kDa activity window was determined using YE, whereas the subsequent mass-spectrometric peptide screening was performed on CA-derived material. Consequently, the peptide-compatible formulas detected by FT-ICR-MS cannot be assumed to account for the activity of the 0.5–1-kDa YE fraction. Other low-molecular-weight constituents of YE, including nucleotide-derived compounds or cofactors, may also contribute and warrant investigation in future studies.

Given the respiratory activity supported by CA, glucose, and potassium, we evaluated whether this compositionally simpler supplementation could reproduce the respiratory response obtained with YE. Although the onset of oxygen depletion was delayed relative to YE, the CA+glucose+potassium treatment reached a similar endpoint by day 7. Thus, within the limits of the present respiration assay, YE could be functionally replaced with respect to the final respiratory response, although respiratory activation occurred more slowly. CA nevertheless remains a chemically complex casein hydrolysate and may vary between manufacturers and production lots. Its use therefore reduces compositional complexity relative to YE but does not constitute a fully defined medium. A genuinely defined formulation will require identification and experimental testing of the individual compounds responsible for the observed respiratory support. No techno-economic assessment was performed in the present study, and CA is not proposed here as an economically preferable industrial substitute for YE. Rather, its value in the present context is to reduce compositional complexity and facilitate the experimental identification of nutritional factors supporting respiration.

FT-ICR-MS analysis of CA-derived cultivation samples provided additional evidence for the chemical diversity of low-molecular-weight compounds potentially compatible with peptide compositions. Screening against the revised in-silico library identified 118 unique elemental formulas compatible with di- or tripeptide compositions across both ionization modes and time points. However, elemental formula and accurate mass do not resolve amino acid sequence order or structural isomers, and these signals therefore represent candidate peptide compositions rather than identified peptide sequences.

Temporal comparison further showed that a subset of peptide-compatible signals disappeared from the inoculated cultures during incubation. After comparison with the uninoculated chemical controls, 28 ionization-mode-specific signals, representing 26 unique molecular formulas, showed culture-associated disappearance (Table 1). This culture-associated disappearance is consistent with microbial utilization or transformation of the corresponding compounds, although their exact biochemical fate cannot be resolved from the present analysis. The presence of oligo-/dipeptide transport systems and aminopeptidases reported in the genome of *Sb. thermosulfidooxidans* strain ST nevertheless provides a plausible physiological basis for the utilization of peptide-bound amino acids by members of this species (Guo et al. 2014).

Analyses of *Alicyclobacillus* sp. SO9 (Kwiecień et al. 2025) and *Sb. thermosulfidooxidans* regarding nutrient requirements and metabolites in the culture liquid by ESI-FT-ICR-MS reveal key differences relevant for bioleaching applications under high-chloride conditions. *Alicyclobacillus* sp. SO9 demonstrates an obligate requirement for NaCl for growth (Huynh et al. 2024) and depends on vitamin supplementation (thiamine and niacin) as well as specific amino acids from the aspartate family (Asp, Met, Thr, Lys) to maintain metabolic activity. Despite supplementation with vitamin mixtures and casamino acids, complete replacement of YE has not been achievable, indicating the presence of additional, unidentified, low-molecular-weight (< 1 kDa) compounds essential for this strain’s growth and activity (Kwiecień et al. 2025). ESI-FT-ICR-MS analyses of YE fractions revealed numerous signals, showing that *Alicyclobacillus* sp. SO9 preferentially takes up guanosine monophosphate, phenylalanine, and tryptophan, but also numerous di- and tripeptides (Kwiecień et al. 2025).

In contrast, *Sb. thermosulfidooxidans* DSM 9293^T^ exhibits halotolerance up to 500 mM NaCl but does not require salt for growth, providing operational flexibility. This strain did not show an apparent requirement for supplementation with the vitamins tested, and under the conditions without additional NaCl examined here, CA, glucose, and potassium supported substantial glucose-dependent respiration. Compared with YE, the respiratory response showed a delayed onset but reached a similar endpoint by day 7. Thus, CA, glucose, and potassium can support a respiratory endpoint comparable to that obtained with YE. FT-ICR-MS analysis of CA-derived cultivation samples identified numerous molecular formulas compatible with candidate di- and tripeptide compositions, including 26 unique formulas showing culture-associated disappearance relative to uninoculated controls. This pattern is consistent with microbial utilization or transformation of the corresponding compounds, although preferential utilization of individual amino acids could not be resolved from the present data. However, CA remains chemically complex and therefore represents a compositionally simpler rather than fully defined supplementation strategy.

The comparative analyses of *Alicyclobacillus* sp. SO9 and *Sb. thermosulfidooxidans* illustrate the diverse strategies these acidophilic iron oxidizers employ to sustain bioleaching activity. The findings broaden the understanding of nutrient requirements and metabolic plasticity among chloride-requiring/chloride-tolerant iron-oxidizing bacteria and thus provide a conceptual basis for further efforts toward developing compositionally simpler cultivation strategies for chloride-tolerant microorganisms relevant to saline bioleaching systems.

## Supplementary Information

Below is the link to the electronic supplementary material.

Supplementary Material 1

## Data Availability

The datasets generated and/or analysed during the current study are available from the corresponding author on reasonable request.
