# Supplementary material for: Peptides, not vitamins: Yeast extract components sustaining glucose respiration of the halotolerant acidophile Sulfobacillus thermosulfidooxidans
Source: World J Microbiol Biotechnol. 2026 Nov 5;42(11):644. doi: 10.1007/s11274-026-05336-8 (PMC13740008; doi:10.1007/s11274-026-05336-8)
Supplement: Supplementary file 1 [file 11274_2026_5336_MOESM1_ESM.docx]

**Supplementary Material for:**

Peptides, not vitamins: yeast extract components sustaining glucose respiration of the halotolerant acidophile Sulfobacillus thermosulfidooxidans

**Katarzyna Kwiecień^1*^**

**Stefan Kaschabek^1^**

**Jan Zuber^2^**

**Michael Schlömann^1^**

**^1^** Environmental Microbiology, Institute of Biosciences, TU Bergakademie Freiberg, Freiberg, Lessingstraße 45*, 09599* Freiberg, Germany

^2^ Institute for Analytical Chemistry, TU Bergakademie Freiberg, Freiberg, Lessingstraße *45, 09599* Freiberg, Germany

*Corresponding author: Katarzyna Kwiecień

Email: [katarzyna.kwiecien@doktorand.tu-freiberg.de](mailto:katarzyna.kwiecien@doktorand.tu-freiberg.de)

b

a

**Online Resource** **1.** Representative assessment of the chloride-tolerance threshold of Sb. harzensis DSM 109850T based on biological Fe(II) oxidation. Biological Fe(II) oxidation remained detectable up to 125 mM NaCl (4.43 g Cl⁻ l⁻¹), whereas no biologically mediated Fe(II) oxidation was detectable at 150 mM NaCl. Sb. harzensis DSM 109850^T^ was cultivated in modified Mackintosh (1978) medium (initial pH 1.8), supplemented with 220 mg l^-1^ CoCl_2_·6 H_2_O, 5.56 g l⁻¹ (20 mM) FeSO₄·7H₂O, 0.2 g l⁻¹ YE, and incubated at 45 °C with shaking at 120 rpm. The inhibitory effect of sodium chloride on microbial iron oxidation was evaluated across various Sulfobacillus strains over a concentration range of 0–1000 mM NaCl.

**Online Resource** **2.** Respiratory response of Sb. thermosulfidooxidans over 7 days of incubation with glucose and various additional supplements (potassium, casamino acids, or a mixture of 6 vitamins). The tested conditions included: K - 0.03 g l⁻¹ K₂SO₄, 2K - 0.06 g l⁻¹ K₂SO₄, 2CA - 0.6 g l⁻¹ CA, 2V – doubled concentrated vitamin mixture, YE - 0.2 g l^-1^ yeast extract, CC – chemical control (without bacteria) supplemented with potassium, Casamino Acids, the mixture of 6 vitamins, and glucose. Data points correspond to the mean of triplicate samples, with variability shown as standard deviation.

**Online Resource Table S1.** Peptide-compatible molecular formulas detected in CA-derived cultivation samples by ESI-FT-ICR-MS. Peptide-compatible molecular formulas detected in inoculated cultures at t0 and/or t7 are shown together with their candidate peptide compositions, ionization mode, detection patterns, and relative ESI signal intensities in the corresponding inoculated cultures and uninoculated chemical controls. Relative ESI signal intensities were normalized to the base-peak intensity of the respective spectrum (base peak = 100) and are considered semi-quantitative. Candidate peptide compositions represent amino acid compositions compatible with the assigned elemental formula and exact mass; sequence order cannot be resolved, and leucine and isoleucine cannot be distinguished.

| **Molecular formula** | **Candidate peptide composition(s)** | **Calculated neutral monoisotopic mass (Da)** | **ESI mode** | **Culture t0 relative ESI intensity (base peak = 100)** | **Culture t7 relative ESI intensity (base peak = 100)** | **CC t0 relative ESI intensity (base peak = 100)** | **CC t7 relative ESI intensity (base peak = 100)** | **Signal pattern** |
| --- | --- | --- | --- | --- | --- | --- | --- | --- |
| C7H14N2O3 | Gly+Val | 174.100442 | ESI(+) | 0.0528 | ND | ND | ND | Loss of detectability in culture; CC comparison incomplete |
| C8H16N2O3 | Ala+Val; Gly+Ile; Gly+Leu | 188.116092 | ESI(+) | 0.0736 | ND | 0.104 | ND | Loss of detectability in both culture and CC |
| C9H18N2O3 | Ala+Ile; Ala+Leu | 202.131742 | ESI(+) | 0.0566 | ND | ND | 0.0852 | Loss of detectability in culture; CC comparison incomplete |
| C10H16N2O3 | Pro+Pro | 212.116092 | ESI(+) | 0.0687 | ND | 0.1218 | ND | Loss of detectability in both culture and CC |
| C10H18N2O3 | Val+Pro | 214.131742 | ESI(+) | 0.8681 | 0.0615 | 1.7491 | 0.7472 | Signal decrease greater in culture than CC |
| C10H18N2O3 | Val+Pro | 214.131742 | ESI(−) | 0.0646 | ND | ND | 0.1631 | Loss of detectability in culture; CC comparison incomplete |
| C10H20N2O3 | Val+Val | 216.147393 | ESI(+) | 0.2358 | 0.0933 | 0.4591 | 0.1932 | Signal decrease greater in culture than CC |
| C9H18N2O4 | Ser+Ile; Ser+Leu; Val+Thr | 218.126657 | ESI(+) | 0.2917 | ND | 0.4984 | 0.1409 | Loss of detectability in culture; retained in CC |
| C9H18N2O4 | Ser+Ile; Ser+Leu; Val+Thr | 218.126657 | ESI(−) | 0.1004 | ND | 0.1196 | 0.242 | Loss of detectability in culture; retained in CC |
| C8H16N2O3S | Ala+Met; Cys+Val | 220.088164 | ESI(+) | ND | 0.0889 | ND | ND | Detected only at t7 |
| C11H20N2O3 | Ile+Pro; Leu+Pro | 228.147393 | ESI(+) | 3.5263 | 0.6475 | 8.8616 | 3.0563 | Signal decrease greater in culture than CC |
| C11H20N2O3 | Ile+Pro; Leu+Pro | 228.147393 | ESI(−) | 0.1779 | 0.6477 | 0.2003 | 0.4807 | Signal increase in culture |
| C11H22N2O3 | Val+Ile; Val+Leu | 230.163043 | ESI(+) | 4.1538 | 0.8169 | 9.3336 | 3.4211 | Signal decrease greater in culture than CC |
| C11H22N2O3 | Val+Ile; Val+Leu | 230.163043 | ESI(−) | 0.5884 | 2.4905 | 0.6058 | 1.6486 | Signal increase in culture |
| C10H20N2O4 | Ile+Thr; Leu+Thr | 232.142307 | ESI(+) | 0.397 | 0.0746 | 0.767 | 0.5512 | Signal decrease greater in culture than CC |
| C10H20N2O4 | Ile+Thr; Leu+Thr | 232.142307 | ESI(−) | 0.1547 | ND | 0.1383 | 0.3503 | Loss of detectability in culture; retained in CC |
| C9H18N2O3S | Cys+Ile; Cys+Leu | 234.103814 | ESI(+) | ND | 0.0584 | ND | ND | Detected only at t7 |
| C8H16N2O4S | Ser+Met | 236.083078 | ESI(+) | 0.0605 | 0.1231 | ND | ND | Signal increase in culture |
| C12H16N2O3 | Ala+Phe | 236.116092 | ESI(+) | 0.0629 | ND | 0.1266 | 0.0937 | Loss of detectability in culture; retained in CC |
| C12H24N2O3 | Ile+Ile; Leu+Ile; Leu+Leu | 244.178693 | ESI(+) | 3.905 | 0.9736 | 8.3475 | 3.749 | Signal decrease greater in culture than CC |
| C12H24N2O3 | Ile+Ile; Leu+Ile; Leu+Leu | 244.178693 | ESI(−) | 0.5282 | 2.8387 | 0.537 | 1.8678 | Signal increase in culture |
| C11H23N3O3 | Val+Lys | 245.173942 | ESI(+) | 0.4483 | ND | 0.7186 | 0.3368 | Loss of detectability in culture; retained in CC |
| C10H18N2O3S | Met+Pro | 246.103814 | ESI(+) | 0.1948 | ND | 0.3897 | 0.0804 | Loss of detectability in culture; retained in CC |
| C10H18N2O5 | Ile+Asp; Leu+Asp; Val+Glu | 246.121572 | ESI(+) | 0.1564 | ND | 0.3287 | ND | Loss of detectability in both culture and CC |
| C10H18N2O5 | Ile+Asp; Leu+Asp; Val+Glu | 246.121572 | ESI(−) | 0.3306 | ND | 0.5693 | 0.2889 | Loss of detectability in culture; retained in CC |
| C10H20N2O3S | Val+Met | 248.119464 | ESI(+) | 0.1613 | 0.0922 | 0.6856 | 0.1081 | Signal decrease equal to or smaller than CC |
| C10H20N2O3S | Val+Met | 248.119464 | ESI(−) | ND | 0.1145 | ND | ND | Detected only at t7 |
| C9H18N2O4S | Thr+Met | 250.098728 | ESI(+) | 0.062 | ND | 0.1383 | ND | Loss of detectability in both culture and CC |
| C12H16N2O4 | Ala+Tyr; Ser+Phe | 252.111007 | ESI(+) | 0.2005 | ND | 0.4181 | 0.1462 | Loss of detectability in culture; retained in CC |
| C12H16N2O4 | Ala+Tyr; Ser+Phe | 252.111007 | ESI(−) | 0.0938 | 0.1016 | 0.1382 | 0.2254 | Signal increase in culture |
| C11H18N4O3 | Val+His | 254.13789 | ESI(+) | 0.0811 | ND | 0.1187 | 0.0709 | Loss of detectability in culture; retained in CC |
| C12H25N3O3 | Ile+Lys; Leu+Lys | 259.189592 | ESI(+) | 2.874 | 1.0132 | 4.8416 | 2.6426 | Signal decrease greater in culture than CC |
| C12H25N3O3 | Ile+Lys; Leu+Lys | 259.189592 | ESI(−) | ND | 0.2412 | ND | 0.128 | Detected only at t7 |
| C11H20N2O5 | Ile+Glu; Leu+Glu | 260.137222 | ESI(+) | 1.0266 | 0.1011 | 2.5346 | 0.619 | Signal decrease greater in culture than CC |
| C11H20N2O5 | Ile+Glu; Leu+Glu | 260.137222 | ESI(−) | 1.9299 | 0.6196 | 4.0705 | 2.5498 | Signal decrease greater in culture than CC |
| C14H18N2O3 | Phe+Pro | 262.131742 | ESI(+) | 3.7522 | 0.742 | 8.6919 | 3.4056 | Signal decrease greater in culture than CC |
| C14H18N2O3 | Phe+Pro | 262.131742 | ESI(−) | 0.2647 | 1.2566 | 0.2521 | 0.8775 | Signal increase in culture |
| C11H22N2O3S | Ile+Met; Leu+Met | 262.135114 | ESI(+) | 0.1019 | ND | 0.4719 | ND | Loss of detectability in both culture and CC |
| C11H22N2O3S | Ile+Met; Leu+Met | 262.135114 | ESI(−) | ND | 0.1126 | ND | ND | Detected only at t7 |
| C14H20N2O3 | Val+Phe | 264.147393 | ESI(+) | 2.1325 | 0.5077 | 4.727 | 2.051 | Signal decrease greater in culture than CC |
| C14H20N2O3 | Val+Phe | 264.147393 | ESI(−) | 0.2568 | 1.1973 | 0.264 | 0.9012 | Signal increase in culture |
| C13H18N2O4 | Thr+Phe | 266.126657 | ESI(+) | 0.0883 | 0.0546 | 0.2114 | 0.1027 | Signal decrease equal to or smaller than CC |
| C12H20N4O3 | Ile+His; Leu+His | 268.153541 | ESI(+) | 2.5865 | 1.4334 | 4.9765 | 2.5588 | Signal decrease equal to or smaller than CC |
| C12H20N4O3 | Ile+His; Leu+His | 268.153541 | ESI(−) | 1.1258 | 1.3887 | 2.4806 | 1.3121 | Signal increase in culture |
| C11H21N5O3 | Arg+Pro | 271.16444 | ESI(+) | 0.31 | 0.0841 | 0.4015 | 0.2902 | Signal decrease greater in culture than CC |
| C11H23N5O3 | Val+Arg | 273.180090 | ESI(+) | 2.2871 | 0.9965 | 3.4156 | 2.0436 | Signal decrease greater in culture than CC |
| C11H23N5O3 | Val+Arg | 273.180090 | ESI(−) | ND | 0.1118 | ND | ND | Detected only at t7 |
| C11H23N3O3S | Met+Lys | 277.146013 | ESI(+) | 0.0847 | ND | 0.1904 | ND | Loss of detectability in both culture and CC |
| C10H18N2O5S | Met+Glu | 278.093643 | ESI(+) | 0.077 | ND | 0.1438 | ND | Loss of detectability in both culture and CC |
| C10H18N2O5S | Met+Glu | 278.093643 | ESI(−) | 0.1024 | ND | 0.1766 | ND | Loss of detectability in both culture and CC |
| C14H18N2O4 | Tyr+Pro | 278.126657 | ESI(+) | 1.3966 | 0.2004 | 3.3843 | 0.9707 | Signal decrease greater in culture than CC |
| C14H18N2O4 | Tyr+Pro | 278.126657 | ESI(−) | 0.1027 | 0.2763 | 0.1372 | 0.2538 | Signal increase in culture |
| C15H22N2O3 | Ile+Phe; Leu+Phe | 278.163043 | ESI(+) | 2.8144 | 0.6562 | 5.0568 | 2.6796 | Signal decrease greater in culture than CC |
| C15H22N2O3 | Ile+Phe; Leu+Phe | 278.163043 | ESI(−) | 0.3731 | 1.735 | 0.287 | 1.2508 | Signal increase in culture |
| C10H20N2O3S2 | Met+Met | 280.091535 | ESI(+) | 0.1871 | 0.1065 | 0.2424 | 0.1041 | Signal decrease equal to or smaller than CC |
| C14H20N2O4 | Val+Tyr | 280.142307 | ESI(+) | 2.1682 | 0.4058 | 4.9023 | 1.6594 | Signal decrease greater in culture than CC |
| C14H20N2O4 | Val+Tyr | 280.142307 | ESI(−) | 0.2453 | 0.8991 | 0.2801 | 0.646 | Signal increase in culture |
| C13H18N2O5 | Thr+Tyr | 282.121572 | ESI(+) | 0.063 | ND | 0.1442 | ND | Loss of detectability in both culture and CC |
| C13H23N3O4 | Ala+Val+Pro; Gly+Ile+Pro; Gly+Leu+Pro | 285.168856 | ESI(+) | 0.1971 | ND | 0.4414 | 0.1726 | Loss of detectability in culture; retained in CC |
| C11H18N4O3S | Met+His | 286.109962 | ESI(+) | 0.0743 | ND | 0.1238 | ND | Loss of detectability in both culture and CC |
| C12H25N5O3 | Ile+Arg; Leu+Arg | 287.195740 | ESI(+) | 6.589 | 5.9673 | 12.9874 | 6.3607 | Signal decrease equal to or smaller than CC |
| C12H25N5O3 | Ile+Arg; Leu+Arg | 287.195740 | ESI(−) | 0.1078 | 0.6617 | 0.1299 | 0.1723 | Signal increase in culture |
| C15H23N3O3 | Phe+Lys | 293.173942 | ESI(+) | 1.157 | 1.1057 | 2.4464 | 1.0703 | Signal decrease equal to or smaller than CC |
| C15H23N3O3 | Phe+Lys | 293.173942 | ESI(−) | ND | 0.226 | ND | ND | Detected only at t7 |
| C14H18N2O5 | Phe+Glu | 294.121572 | ESI(+) | 0.1196 | ND | 0.3271 | 0.1061 | Loss of detectability in culture; retained in CC |
| C14H18N2O5 | Phe+Glu | 294.121572 | ESI(−) | 0.1713 | 0.156 | 0.4734 | 0.2174 | Signal decrease equal to or smaller than CC |
| C15H22N2O4 | Ile+Tyr; Leu+Tyr | 294.157957 | ESI(+) | 4.1689 | 0.8142 | 8.1713 | 3.3087 | Signal decrease greater in culture than CC |
| C15H22N2O4 | Ile+Tyr; Leu+Tyr | 294.157957 | ESI(−) | 0.5623 | 2.2496 | 0.5434 | 1.5848 | Signal increase in culture |
| C11H21N5O5 | Glu+Arg | 303.154269 | ESI(+) | 0.2052 | ND | 0.3503 | ND | Loss of detectability in both culture and CC |
| C13H25N3O5 | Ala+Ile+Thr; Ala+Leu+Thr; Ser+Val+Val | 303.179421 | ESI(+) | 0.0984 | ND | 0.172 | 0.1123 | Loss of detectability in culture; retained in CC |
| C15H23N3O4 | Pro+Pro+Pro; Tyr+Lys | 309.168856 | ESI(+) | 1.3867 | 0.744 | 2.599 | 1.2003 | Signal decrease equal to or smaller than CC |
| C15H23N3O4 | Pro+Pro+Pro; Tyr+Lys | 309.168856 | ESI(−) | ND | 0.2172 | ND | 0.0925 | Detected only at t7 |
| C14H18N2O6 | Tyr+Glu | 310.116486 | ESI(+) | 0.1339 | ND | 0.4777 | 0.1177 | Loss of detectability in culture; retained in CC |
| C14H18N2O6 | Tyr+Glu | 310.116486 | ESI(−) | 0.2981 | 0.2186 | 0.6772 | 0.4205 | Signal decrease equal to or smaller than CC |
| C15H25N3O4 | Val+Pro+Pro | 311.184506 | ESI(+) | 0.1285 | ND | ND | 0.1036 | Loss of detectability in culture; CC comparison incomplete |
| C14H20N2O4S | Met+Tyr | 312.114378 | ESI(+) | 0.0565 | ND | 0.2107 | 0.072 | Loss of detectability in culture; retained in CC |
| C18H20N2O3 | Phe+Phe | 312.147393 | ESI(+) | 0.2306 | ND | 0.4423 | 0.223 | Loss of detectability in culture; retained in CC |
| C18H20N2O3 | Phe+Phe | 312.147393 | ESI(−) | ND | 0.2035 | ND | 0.1057 | Detected only at t7 |
| C15H27N3O4 | Val+Val+Pro | 313.200156 | ESI(+) | 0.2521 | 0.0605 | 0.53 | 0.2316 | Signal decrease greater in culture than CC |
| C14H25N3O5 | Ser+Ile+Pro; Ser+Leu+Pro; Val+Thr+Pro | 315.179421 | ESI(+) | 0.5827 | ND | 1.2936 | 0.4636 | Loss of detectability in culture; retained in CC |
| C14H25N3O5 | Ser+Ile+Pro; Ser+Leu+Pro; Val+Thr+Pro | 315.179421 | ESI(−) | 0.0855 | 0.1724 | 0.1291 | 0.2197 | Signal increase in culture |
| C15H29N3O4 | Ala+Ile+Ile; Ala+Leu+Ile; Ala+Leu+Leu; Val+Val+Val | 315.215806 | ESI(+) | 0.9442 | 0.2792 | 2.1395 | 0.8692 | Signal decrease greater in culture than CC |
| C15H29N3O4 | Ala+Ile+Ile; Ala+Leu+Ile; Ala+Leu+Leu; Val+Val+Val | 315.215806 | ESI(−) | 0.1367 | 0.6859 | 0.1619 | ND | Signal increase in culture |
| C14H27N3O5 | Ser+Val+Ile; Ser+Val+Leu; Val+Val+Thr | 317.195071 | ESI(+) | 0.4398 | 0.0954 | 1.1018 | 0.3746 | Signal decrease greater in culture than CC |
| C14H27N3O5 | Ser+Val+Ile; Ser+Val+Leu; Val+Val+Thr | 317.195071 | ESI(−) | 0.2969 | 0.8698 | 0.5857 | 0.7151 | Signal increase in culture |
| C13H25N3O4S | Ala+Val+Met; Cys+Val+Val; Gly+Ile+Met; Gly+Leu+Met | 319.156577 | ESI(+) | 0.1159 | 0.0532 | 0.2618 | ND | Signal decrease in culture; CC comparison incomplete |
| C13H25N3O6 | Ser+Ile+Thr; Ser+Leu+Thr; Val+Thr+Thr | 319.174336 | ESI(+) | 0.0587 | ND | ND | ND | Loss of detectability in culture; CC comparison incomplete |
| C15H23N5O3 | Phe+Arg | 321.180090 | ESI(+) | 2.7679 | 2.4286 | 4.9918 | 2.6236 | Signal decrease equal to or smaller than CC |
| C15H23N5O3 | Phe+Arg | 321.180090 | ESI(−) | 0.0651 | 0.3042 | ND | 0.1011 | Signal increase in culture |
| C16H27N3O4 | Ile+Pro+Pro; Leu+Pro+Pro | 325.200156 | ESI(+) | 1.1959 | 0.4109 | 2.7484 | 1.1151 | Signal decrease greater in culture than CC |
| C16H27N3O4 | Ile+Pro+Pro; Leu+Pro+Pro | 325.200156 | ESI(−) | ND | 0.3042 | ND | 0.162 | Detected only at t7 |
| C16H29N3O4 | Val+Ile+Pro; Val+Leu+Pro | 327.215806 | ESI(+) | 2.7252 | 0.8287 | 5.7702 | 2.7601 | Signal decrease greater in culture than CC |
| C16H29N3O4 | Val+Ile+Pro; Val+Leu+Pro | 327.215806 | ESI(−) | ND | 0.9508 | 0.2002 | 0.5975 | Detected only at t7 |
| C18H20N2O4 | Phe+Tyr | 328.142307 | ESI(+) | 1.183 | ND | 2.2639 | 1.0026 | Loss of detectability in culture; retained in CC |
| C18H20N2O4 | Phe+Tyr | 328.142307 | ESI(−) | 0.1889 | 0.7608 | 0.1865 | 0.591 | Signal increase in culture |
| C15H27N3O5 | Ile+Thr+Pro; Leu+Thr+Pro | 329.195071 | ESI(+) | 0.0835 | ND | 0.1255 | ND | Loss of detectability in both culture and CC |
| C16H31N3O4 | Val+Val+Ile; Val+Val+Leu | 329.231456 | ESI(+) | 0.0615 | ND | 0.1065 | ND | Loss of detectability in both culture and CC |
| C15H29N3O5 | Ser+Ile+Ile; Ser+Leu+Ile; Ser+Leu+Leu; Val+Ile+Thr; Val+Leu+Thr | 331.210721 | ESI(+) | 0.474 | 0.1957 | 1.1096 | 0.4349 | Signal decrease equal to or smaller than CC |
| C15H29N3O5 | Ser+Ile+Ile; Ser+Leu+Ile; Ser+Leu+Leu; Val+Ile+Thr; Val+Leu+Thr | 331.210721 | ESI(−) | ND | 0.7601 | 0.4205 | 0.5408 | Detected only at t7 |
| C13H23N3O7 | Ser+Ile+Asp; Ser+Leu+Asp; Ser+Val+Glu; Val+Thr+Asp | 333.153600 | ESI(−) | 0.063 | ND | ND | ND | Loss of detectability in culture; CC comparison incomplete |
| C13H25N3O5S | Cys+Ile+Thr; Cys+Leu+Thr; Ser+Val+Met | 335.151492 | ESI(+) | 0.0563 | ND | 0.1324 | 0.1117 | Loss of detectability in culture; retained in CC |
| C17H25N3O4 | Ala+Val+Phe; Gly+Ile+Phe; Gly+Leu+Phe | 335.184506 | ESI(+) | 0.1233 | 0.0644 | 0.2608 | 0.0967 | Signal decrease equal to or smaller than CC |
| C15H23N5O4 | Tyr+Arg | 337.175004 | ESI(+) | 1.8873 | 1.629 | 3.5285 | 1.683 | Signal decrease equal to or smaller than CC |
| C15H23N5O4 | Tyr+Arg | 337.175004 | ESI(−) | ND | 0.1964 | ND | ND | Detected only at t7 |
| C17H31N3O4 | Ile+Ile+Pro; Leu+Ile+Pro; Leu+Leu+Pro | 341.231456 | ESI(+) | 2.6239 | 0.7669 | 5.079 | 2.8238 | Signal decrease greater in culture than CC |
| C17H31N3O4 | Ile+Ile+Pro; Leu+Ile+Pro; Leu+Leu+Pro | 341.231456 | ESI(−) | 0.2027 | 1.1363 | 0.1852 | 0.7579 | Signal increase in culture |
| C16H30N4O4 | Val+Lys+Pro | 342.226705 | ESI(+) | 0.1201 | 0.0537 | 0.1871 | 0.0925 | Signal decrease greater in culture than CC |
| C15H25N3O6 | Ile+Asp+Pro; Leu+Asp+Pro; Val+Glu+Pro | 343.174336 | ESI(+) | 0.2081 | ND | 0.4796 | 0.1395 | Loss of detectability in culture; retained in CC |
| C15H25N3O6 | Ile+Asp+Pro; Leu+Asp+Pro; Val+Glu+Pro | 343.174336 | ESI(−) | 0.1429 | 0.1453 | 0.3028 | 0.2233 | Signal increase in culture |
| C17H33N3O4 | Val+Ile+Ile; Val+Leu+Ile; Val+Leu+Leu | 343.247107 | ESI(+) | 1.8405 | 0.5574 | 3.5115 | 2.0143 | Signal decrease greater in culture than CC |
| C17H33N3O4 | Val+Ile+Ile; Val+Leu+Ile; Val+Leu+Leu | 343.247107 | ESI(−) | 0.225 | 1.3925 | 0.259 | 0.9619 | Signal increase in culture |
| C18H20N2O5 | Tyr+Tyr | 344.137222 | ESI(+) | 0.3277 | ND | 0.7451 | ND | Loss of detectability in both culture and CC |
| C18H20N2O5 | Tyr+Tyr | 344.137222 | ESI(−) | ND | 0.2062 | ND | 0.1805 | Detected only at t7 |
| C15H27N3O6 | Val+Ile+Asp; Val+Leu+Asp; Val+Val+Glu | 345.189986 | ESI(−) | 0.0714 | ND | 0.1276 | 0.0964 | Loss of detectability in culture; retained in CC |
| C16H31N3O5 | Ile+Ile+Thr; Leu+Ile+Thr; Leu+Leu+Thr | 345.226371 | ESI(+) | ND | 0.0557 | 0.1085 | 0.065 | Detected only at t7 |
| C15H30N4O5 | Ser+Ile+Lys; Ser+Leu+Lys; Val+Thr+Lys | 346.221620 | ESI(+) | 0.0976 | ND | 0.1334 | 0.0972 | Loss of detectability in culture; retained in CC |
| C14H25N3O7 | Ile+Thr+Asp; Leu+Thr+Asp; Ser+Ile+Glu; Ser+Leu+Glu; Val+Thr+Glu | 347.169250 | ESI(−) | 0.1898 | ND | 0.3791 | ND | Loss of detectability in both culture and CC |
| C14H27N3O5S | Ser+Ile+Met; Ser+Leu+Met; Val+Thr+Met | 349.167142 | ESI(+) | 0.4255 | 0.2584 | 1.0832 | 0.1178 | Signal decrease equal to or smaller than CC |
| C14H27N3O5S | Ser+Ile+Met; Ser+Leu+Met; Val+Thr+Met | 349.167142 | ESI(−) | 0.4862 | 0.6969 | 1.0616 | 0.3162 | Signal increase in culture |
| C12H23N3O5S2 | Cys+Thr+Met | 353.107913 | ESI(+) | 0.0915 | ND | 0.1851 | 0.0611 | Loss of detectability in culture; retained in CC |
| C12H23N3O5S2 | Cys+Thr+Met | 353.107913 | ESI(−) | 0.0948 | 0.1732 | 0.2311 | 0.1701 | Signal increase in culture |
| C17H32N4O4 | Ile+Lys+Pro; Leu+Lys+Pro | 356.242356 | ESI(+) | 0.2602 | 0.2831 | 0.4936 | 0.2755 | Signal increase in culture |
| C16H27N3O6 | Ile+Glu+Pro; Leu+Glu+Pro | 357.189986 | ESI(+) | 0.3012 | ND | 0.7984 | 0.184 | Loss of detectability in culture; retained in CC |
| C16H27N3O6 | Ile+Glu+Pro; Leu+Glu+Pro | 357.189986 | ESI(−) | 0.2101 | ND | 0.6082 | 0.3076 | Loss of detectability in culture; retained in CC |
| C17H34N4O4 | Val+Ile+Lys; Val+Leu+Lys | 358.258006 | ESI(+) | 0.8638 | 1.0652 | 2.0374 | 0.9555 | Signal increase in culture |
| C17H34N4O4 | Val+Ile+Lys; Val+Leu+Lys | 358.258006 | ESI(−) | ND | 0.2509 | ND | ND | Detected only at t7 |
| C19H25N3O4 | Phe+Pro+Pro | 359.184506 | ESI(+) | 0.2271 | ND | ND | 0.225 | Loss of detectability in culture; CC comparison incomplete |
| C19H25N3O4 | Phe+Pro+Pro | 359.184506 | ESI(−) | ND | 0.0923 | ND | ND | Detected only at t7 |
| C16H29N3O4S | Ile+Met+Pro; Leu+Met+Pro | 359.187878 | ESI(+) | 0.3801 | 0.0883 | 0.1383 | 0.0998 | Signal decrease greater in culture than CC |
| C16H29N3O4S | Ile+Met+Pro; Leu+Met+Pro | 359.187878 | ESI(−) | ND | 0.1619 | ND | ND | Detected only at t7 |
| C16H29N3O6 | Ile+Ile+Asp; Leu+Ile+Asp; Leu+Leu+Asp; Val+Ile+Glu; Val+Leu+Glu | 359.205636 | ESI(+) | 0.734 | 0.0536 | 2.2583 | 0.6743 | Signal decrease greater in culture than CC |
| C16H29N3O6 | Ile+Ile+Asp; Leu+Ile+Asp; Leu+Leu+Asp; Val+Ile+Glu; Val+Leu+Glu | 359.205636 | ESI(−) | 1.3215 | 1.8152 | 3.9096 | 2.0406 | Signal increase in culture |
| C16H32N4O5 | Ile+Thr+Lys; Leu+Thr+Lys | 360.237270 | ESI(+) | 0.157 | 0.0957 | 0.3262 | 0.1581 | Signal decrease equal to or smaller than CC |
| C15H27N3O7 | Ile+Thr+Glu; Leu+Thr+Glu | 361.184900 | ESI(+) | 0.1297 | ND | 0.1353 | ND | Loss of detectability in both culture and CC |
| C15H27N3O7 | Ile+Thr+Glu; Leu+Thr+Glu | 361.184900 | ESI(−) | 0.2132 | 0.1947 | 0.5159 | 0.3437 | Signal decrease equal to or smaller than CC |
| C17H27N5O4 | Ile+His+Pro; Leu+His+Pro | 365.206304 | ESI(+) | 0.3636 | 0.4682 | 0.8149 | 0.4157 | Signal increase in culture |
| C17H27N5O4 | Ile+His+Pro; Leu+His+Pro | 365.206304 | ESI(−) | 0.0618 | 0.201 | 0.2207 | 0.0951 | Signal increase in culture |
| C17H25N3O4S | Ala+Met+Phe; Cys+Val+Phe | 367.156577 | ESI(+) | ND | 0.0567 | ND | ND | Detected only at t7 |
| C16H30N6O4 | Val+Arg+Pro | 370.232853 | ESI(+) | 0.4688 | ND | 0.5591 | ND | Loss of detectability in both culture and CC |
| C18H36N4O4 | Ile+Ile+Lys; Leu+Ile+Lys; Leu+Leu+Lys | 372.273656 | ESI(+) | 0.1114 | 0.0992 | 0.207 | 0.1099 | Signal decrease equal to or smaller than CC |
| C17H31N3O6 | Ile+Ile+Glu; Leu+Ile+Glu; Leu+Leu+Glu | 373.221286 | ESI(+) | 0.1572 | ND | 0.4093 | 0.1156 | Loss of detectability in culture; retained in CC |
| C17H31N3O6 | Ile+Ile+Glu; Leu+Ile+Glu; Leu+Leu+Glu | 373.221286 | ESI(−) | ND | 0.4158 | 0.5321 | ND | Detected only at t7 |
| C19H25N3O5 | Tyr+Pro+Pro | 375.179421 | ESI(+) | 0.095 | ND | ND | ND | Loss of detectability in culture; CC comparison incomplete |
| C20H29N3O4 | Ile+Phe+Pro; Leu+Phe+Pro | 375.215806 | ESI(+) | 0.1137 | ND | 0.2387 | 0.1016 | Loss of detectability in culture; retained in CC |
| C17H20N4O6 | Gly+Trp+Asp | 376.138284 | ESI(−) | ND | 0.1311 | ND | ND | Detected only at t7 |
| C19H27N3O5 | Val+Tyr+Pro | 377.195071 | ESI(+) | 0.5062 | 0.1185 | 1.2085 | 0.4462 | Signal decrease greater in culture than CC |
| C19H27N3O5 | Val+Tyr+Pro | 377.195071 | ESI(−) | 0.1095 | 0.3492 | 0.1324 | 0.2962 | Signal increase in culture |
| C18H27N3O6 | Ser+Ile+Tyr; Ser+Leu+Tyr; Val+Thr+Tyr | 381.189986 | ESI(+) | 0.2293 | ND | 0.6221 | ND | Loss of detectability in both culture and CC |
| C18H27N3O6 | Ser+Ile+Tyr; Ser+Leu+Tyr; Val+Thr+Tyr | 381.189986 | ESI(−) | 0.1679 | 0.4359 | ND | 0.3261 | Signal increase in culture |
| C18H31N5O4 | Ile+Ile+His; Leu+Ile+His; Leu+Leu+His | 381.237604 | ESI(+) | 0.1241 | 0.0516 | ND | ND | Signal decrease in culture; CC comparison incomplete |
| C17H32N6O4 | Ile+Arg+Pro; Leu+Arg+Pro | 384.248504 | ESI(+) | 0.2341 | 0.3257 | 0.4722 | ND | Signal increase in culture |
| C17H34N6O4 | Val+Ile+Arg; Val+Leu+Arg | 386.264154 | ESI(+) | ND | 0.0997 | ND | ND | Detected only at t7 |
| C17H32N4O6 | Ile+Glu+Lys; Leu+Glu+Lys | 388.232185 | ESI(+) | 0.3254 | 0.0912 | 0.5788 | 0.0981 | Signal decrease equal to or smaller than CC |
| C17H32N4O6 | Ile+Glu+Lys; Leu+Glu+Lys | 388.232185 | ESI(−) | ND | 0.2507 | 0.5001 | ND | Detected only at t7 |
| C16H32N6O5 | Asn+Lys+Lys; Ile+Thr+Arg; Leu+Thr+Arg | 388.243418 | ESI(+) | 1.5829 | 0.0761 | 2.5468 | ND | Signal decrease in culture; CC comparison incomplete |
| C20H30N4O4 | Phe+Lys+Pro | 390.226705 | ESI(+) | ND | 0.2138 | 0.3437 | 0.1855 | Detected only at t7 |
| C19H25N3O6 | Phe+Glu+Pro | 391.174336 | ESI(−) | 0.0617 | 0.1493 | 0.1984 | 0.1178 | Signal increase in culture |
| C19H27N3O6 | Ile+Phe+Asp; Leu+Phe+Asp; Val+Phe+Glu | 393.189986 | ESI(−) | 0.0766 | 0.1394 | 0.2007 | 0.127 | Signal increase in culture |
| C20H31N3O5 | Val+Ile+Tyr; Val+Leu+Tyr | 393.226371 | ESI(+) | 0.2673 | 0.0557 | 0.5625 | ND | Signal decrease in culture; CC comparison incomplete |
| C20H31N3O5 | Val+Ile+Tyr; Val+Leu+Tyr | 393.226371 | ESI(−) | 0.1028 | 0.3873 | 0.1507 | 0.3017 | Signal increase in culture |
| C17H27N5O6 | Ile+Glu+His; Leu+Glu+His | 397.196134 | ESI(+) | 0.201 | ND | 0.5529 | 0.2214 | Loss of detectability in culture; retained in CC |
| C17H27N5O6 | Ile+Glu+His; Leu+Glu+His | 397.196134 | ESI(−) | 0.1745 | 0.3003 | 0.5555 | 0.2324 | Signal increase in culture |
| C15H21N5O8 | Asp+Glu+His | 399.139013 | ESI(+) | ND | 0.2833 | ND | ND | Detected only at t7 |
| C15H21N5O8 | Asp+Glu+His | 399.139013 | ESI(−) | ND | 0.7301 | ND | ND | Detected only at t7 |
| C18H36N6O4 | Ile+Ile+Arg; Leu+Ile+Arg; Leu+Leu+Arg | 400.279804 | ESI(+) | 0.3669 | ND | 0.455 | 0.3652 | Loss of detectability in culture; retained in CC |
| C19H25N3O7 | Tyr+Glu+Pro | 407.169250 | ESI(−) | ND | 0.1519 | 0.1696 | 0.1048 | Detected only at t7 |
| C20H29N3O6 | Ile+Phe+Glu; Leu+Phe+Glu | 407.205636 | ESI(−) | ND | 0.1072 | 0.1259 | 0.1374 | Detected only at t7 |
| C20H32N4O5 | Val+Tyr+Lys | 408.237270 | ESI(+) | 0.0974 | 0.0958 | 0.1976 | 0.0744 | Signal decrease equal to or smaller than CC |
| C21H34N4O5 | Ile+Tyr+Lys; Leu+Tyr+Lys | 422.252920 | ESI(+) | 0.0829 | 0.051 | 0.1335 | 0.0734 | Signal decrease equal to or smaller than CC |
| C20H29N3O7 | Ile+Tyr+Glu; Leu+Tyr+Glu | 423.200550 | ESI(−) | 0.1127 | 0.1749 | 0.2108 | 0.1681 | Signal increase in culture |
| C19H24N6O6 | Trp+Asn+Asn; Tyr+Asn+His | 432.175733 | ESI(+) | 0.071 | ND | ND | ND | Loss of detectability in culture; CC comparison incomplete |
| C21H34N6O4 | Ile+Phe+Arg; Leu+Phe+Arg | 434.264154 | ESI(+) | ND | 0.0767 | ND | ND | Detected only at t7 |
| C20H32N6O5 | Val+Tyr+Arg | 436.243418 | ESI(+) | 0.3662 | ND | ND | ND | Loss of detectability in culture; CC comparison incomplete |
| C21H34N6O5 | Ile+Tyr+Arg; Leu+Tyr+Arg | 450.259068 | ESI(+) | 0.232 | ND | ND | ND | Loss of detectability in culture; CC comparison incomplete |
